# Solvatochromism and Redox Multi‐Switch in a Trinuclear Cobalt(II) Complex

**DOI:** 10.1002/chem.202501455

**Published:** 2025-07-07

**Authors:** Solène Delaporte, Nathalie Bridonneau, François Lambert, Régis Guillot, Nicolas Suaud, Nathalie Guihéry, Ganping Wang, Thayalan Rajeshkumar, Laurent Maron, Talal Mallah

**Affiliations:** ^1^ Institut de Chimie Moléculaire et des Matériaux d'Orsay CNRS Université Paris‐Saclay UMR 8182 17, avenue des Sciences Orsay 91400 France; ^2^ Laboratoire de Chimie et Physique Quantiques (LCPQ) Université de Toulouse CNRS, 118 route de Narbonne Toulouse F‐31062 France; ^3^ INSA CNRS Université de Toulouse 135, avenue de Rangueil Toulouse F‐31077 France

**Keywords:** hexahydroxytriphenylene, redox, solvatochromism

## Abstract

A trinuclear cobalt(II) complex incorporating the redox‐active hexahydroxytriphenylene (H_6_HHTP) ligand was prepared and isolated in two different redox states: [Co^II^_3_(sq‐sq‐sq)]^3+^ (**1**) and [Co^II^_3_(cat‐sq‐sq)]^2+^ (**2**) (sq = semiquinone; cat = catecholate), enabled by its remarkable solvatochromic behavior. The ligand field tuning of the Co(II) centers through the ancillary ligand Me_3_TPA (tris(6‐methyl‐2‐pyridylmethyl)amine) allowed accessing six reversible one‐electron processes instead of only three with the parent TPA ligand, therefore increasing the range of redox‐coupled magnetic and optical switching in this system. Upon reduction, the three redox processes are ligand‐centered and involve the three sq^•−^/cat^2−^ couples of hexahydroxytriphenylene (HHTP), while we hypothesize that some of the oxidation processes may involve the Co(II) metallic species.

## Graphical abstract

A trinuclear cobalt(II) complex incorporating the redox‐active H_6_HHTP ligand is isolated in two redox states: [CoII3(sq‐sq‐sq)]^3+^ and [CoII3(cat‐sq‐sq)]^2+^ (sq = semiquinone; cat = catecholate), enabled by its remarkable solvatochromic behavior. The ligand field tuning of the Co(II) centers through the ancillary ligand allows accessing six reversible one‐electron processes, increasing the range of redox‐coupled magnetic and optical switching in this system.

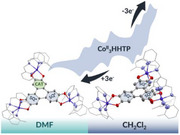

## 1 Introduction

Modulating the physical properties (magnetic, optical) of coordination complexes upon the use of light, temperature, pressure, electron or proton transfer, as well as solvent interaction, is of interest for diverse applications.^[^ ^1^ ^]^ Redox tuning is particularly appealing because one may ultimately inject/remove electrons in cascade, controlling the spin state (for example) at the single‐molecule level. Examples of transition metal^[^ ^2^, ^3^, ^4^, ^5^, ^6^, ^7^, ^8^, ^9^ ^]^ and lanthanide^[^ ^1^, ^10^, ^11^, ^12^, ^13^, ^14^, ^15^ ^]^ ions containing complexes with *multiple* redox states have been reported. Using such systems for any kind of application (such as sensors, for instance) requires full conversion of the molecules from one state to another as well as reversibility of the processes: the redox potential of each state must be reachable without compromising the integrity of the molecule (decoordination, decomposition). But the isolation of the different stable states, required for understanding the magnetic/optical/redox behavior interplay, remains a challenge. Indeed, single‐crystal XRD characterizations in order to study the local evolution of the coordination spheres of the metallic ions upon redox changes are necessary to rationalize the observed effects, by theoretical calculations for example.

**Scheme 1:**
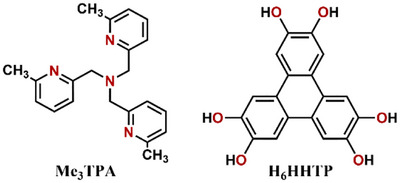
Chemical formula of Me_3_TPA and H_6_HHTP.

The hexahydroxytriphenylene (H_6_HHTP) ligand (Scheme 1) presents, when deprotonated, three bidentate coordination sites and potentially seven accessible electronic states, from triscatecholate (cat‐cat‐cat)^6−^ to trissemiquinone (sq‐sq‐sq)^3−^, and trisquinone (q‐q‐q). We^[^ ^4^, ^9^, ^16^, ^17^ ^]^ and others^[^ ^5^, ^7^, ^8^, ^18^ ^]^ have shown that this redox‐active bridging ligand forms discrete trinuclear magnetic complexes, electrically conductive 2D and 3D MOFs,^[^ ^19^, ^20^ ^]^ as well as molecular cages.^[^ ^21^, ^22^ ^]^ Combining HHTP with Co(II) capped by the tridentate HBTp^(PhPh)^ ligand leads to a trinuclear complex with Co at the oxidation state II and HHTP in the (sq‐sq‐sq)^3−^ state, with four ligand‐centered one‐electron reversible redox processes (three in reduction, sq^•−^/cat^2−^, one in oxidation, sq^•−^/q).^[^ ^9^ ^]^ In this complex, Co(II) is stabilized over Co(III) thanks to pentacoordination that imposes a rather weak ligand field.^[^ ^9^ ^]^ Using the tetradentate tris(2‐pyridylmethyl)amine (TPA) as capping ligand for Co, Suenaga et al. reported the isolation of [Co^III^_3_(TPA)_3_(HHTP)](BF_4_)_4_,^[^ ^18^ ^]^ with HHTP in the (cat‐cat‐sq)^5−^ state exhibiting three one‐electron oxidation waves corresponding to the cat^2−^/sq^•−^ couples. TPA is known to stabilize the low‐spin (LS) Co(III)‐cat state in valence tautomeric complexes because of its rather strong ligand field.^[^ ^23^, ^24^, ^25^, ^26^ ^]^ The introduction of methyl groups at the 6‐position of the pyridine rings (Me_3_TPA, Scheme 1) reduces the Co ligand field and favors the Co^II^_HS_‐sq state.^[^ ^23^, ^24^, ^25^, ^27^, ^28^, ^29^ ^]^

**Figure 1:**
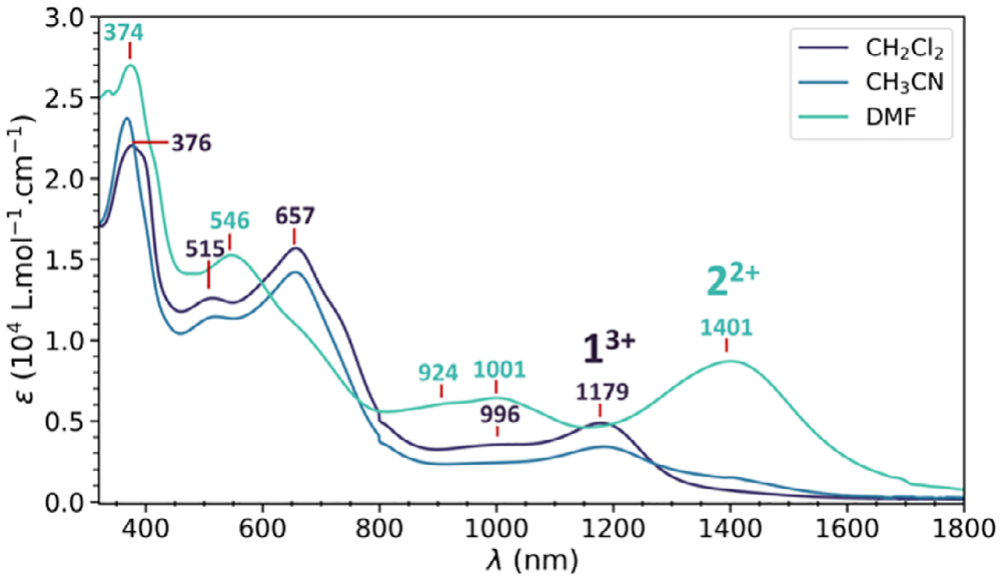
UV‐vis near IR spectra of the [Co_3_(Me_3_TPA)_3_(HHTP)](ClO_4_)_2‐3_ complex in various solvents. The polarity increase from DCM to DMF promotes the stability of **1^3+^** and **2^2+^** in solution, respectively.

Therefore, we anticipated that Me_3_TPA, an ancillary ligand with a ligand field intermediate between HBTp^(PhPh)^ and TPA, could enable accessing trinuclear complexes with rich redox and optical features. Herein, we present the isolation of two complexes, [Co^II^_3_(Me_3_TPA)_3_(HHTP)](ClO_4_)_n_ (n = 3, 2), bearing the central HHTP ligand in two different redox states, (sq‐sq‐sq)^3−^ (**1**) and (cat‐sq‐sq)^4−^ (**2**), for n = 3 and 2, respectively. **1** and **2** were isolated following the procedure summarized below (see Supporting Information for details). The slow addition of a methanolic solution of HHTP^6−^ into a mixture of Co^II^(ClO_4_)_2_·6H_2_O and Me_3_TPA leads to a dark blue precipitate. The precipitate shows solvent‐dependent electronic spectra in the UV‐Vis Near‐IR regions (Figure 1). The spectrum in dimethylformamide (DMF) has an intense band centered at 1401 nm absent in dichloromethane (DCM), while the band at 1179 nm observed in DCM is absent in DMF. In acetonitrile (CH_3_CN), the electronic spectrum seems to correspond to a mixture of the species present in the other two solvents, with a majority of **1**. This solvatochromic effect is already known, the polar solvent stabilizes the species with the largest charge,^[^ ^30^, ^31^, ^32^ ^]^ that is the (cat‐sq‐sq)^4−^ state of HHTP in the present case (even if it leads to a complex of weaker overall charge). The bands at 1179 and 1401 nm have been previously assumed to be characteristic of the (sq‐sq‐sq)^3−^ and the (cat‐sq‐sq)^4−^ states of HHTP, respectively.^[^ ^7^, ^9^, ^16^ ^]^ It is worth noting that, in DMF, the two bands at 924 and 1001 nm can be ascribed to the (cat‐cat‐sq)^5−^ state, revealing a mixture of states, even though **2** is the major species in DMF (Figure S2).

**Figure 2:**
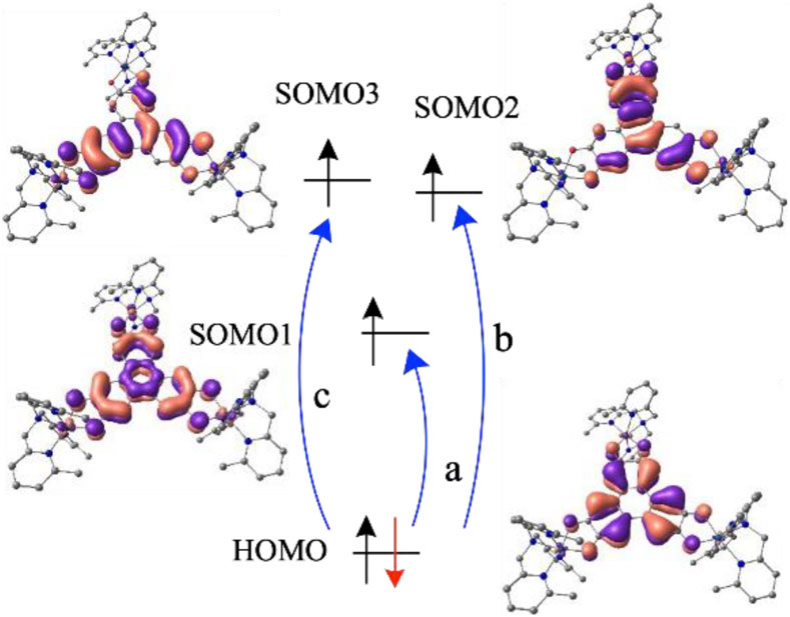
Representation of the excitations responsible for the transitions observed at 1166 nm (a) and 660 nm (b+c) for complex **1**.

To ensure that this is indeed the case, we carried out time‐dependent density functional theory (TD‐DFT) calculations (B3PW91 including solvent effects using solvent‐dependent model, see Supporting Information for details). In all cases, the high spin state (high spin on the ligand ferromagnetically coupled with the Co(II) centers) is found to be the ground state in solution, with the low spin on the ligand being more than 35 kcal/mol higher in energy. On the optimized geometry in solution, the UV‐Visible‐Near IR spectra were simulated for complexes **1** and **2** in solution. Very low‐intensity peaks are found in both cases above 2000 nm and correspond to ligand‐to‐metal charge transfer (LMCT) excitation from the doubly occupied ligand‐based HOMO (Figure S3) to the Co‐based SOMOs. For complex **1** (Figure S4), two distinct bands around 664 nm and 1166 nm are observed and are both associated with ligand‐to‐ligand charge transfer (LLCT) (Figure 2). The band at 664 nm is due to the presence of two states (661 and 680 nm) as depicted in Figure 2. The peak at 1166 nm corresponds to an excitation from the HOMO to the SOMO1. This is in line with the fact that this ligand‐based SOMO1 is the one that got oxidized while going from complex **2** to complex **1** in our computational protocol. For complex **2** (Figures S5, S6), the band at 1400 nm is due to excitations from the doubly occupied HOMO to the two ligand‐based SOMOs (Figure S7), which are very close in energy.

Based on this theoretical analysis, we are confident that the two complexes [Co^II^_3_(Me_3_TPA)_3_(HHTP)]^3+^ and [Co^II^_3_(Me_3_TPA)_3_(HHTP)]^2+^ are stable in DCM and DMF, respectively. Consequently, we carried out the preparation of **1** and **2** in the corresponding solvents and obtained crystals suitable for X‐ray single crystal diffraction (see Supporting Information for the details of the synthetic procedures). The full analysis of the Infrared spectra of **1** and **2** (solid state) and related complexes (Figure S8, Table S1) is consistent with the nature of the oxidation state of HHTP deduced from the electronic spectra.

## 2 Results and Discussion

**Figure 3:**
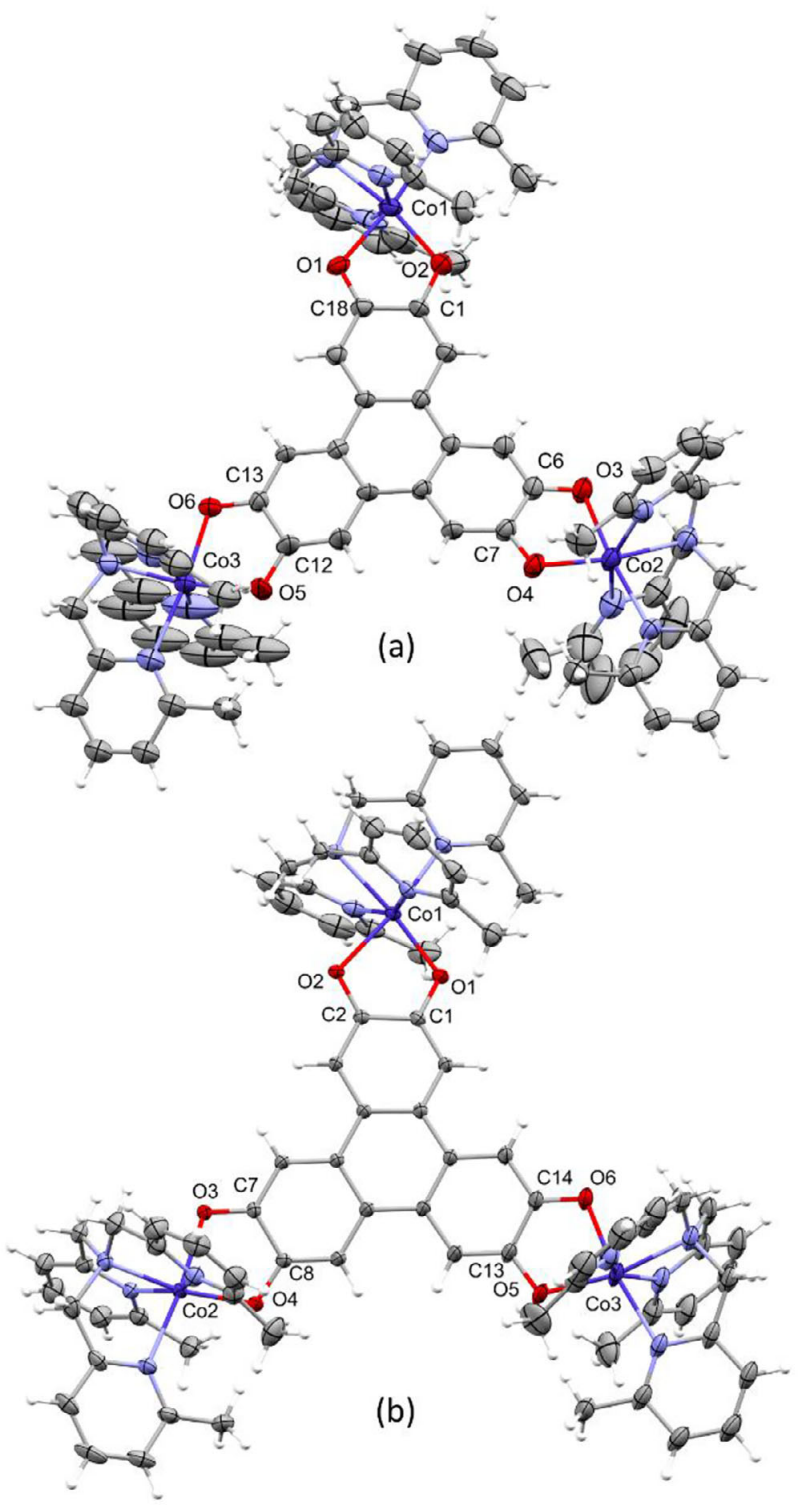
X‐ray crystal structures of a) **1** and b) **2** collected at 200K. Thermal ellipsoids were set at 30% probability level except for hydrogen. Cocrystallized solvent molecules and anions were omitted for clarity. Co blue, N light blue, O red.

**Scheme 2:**
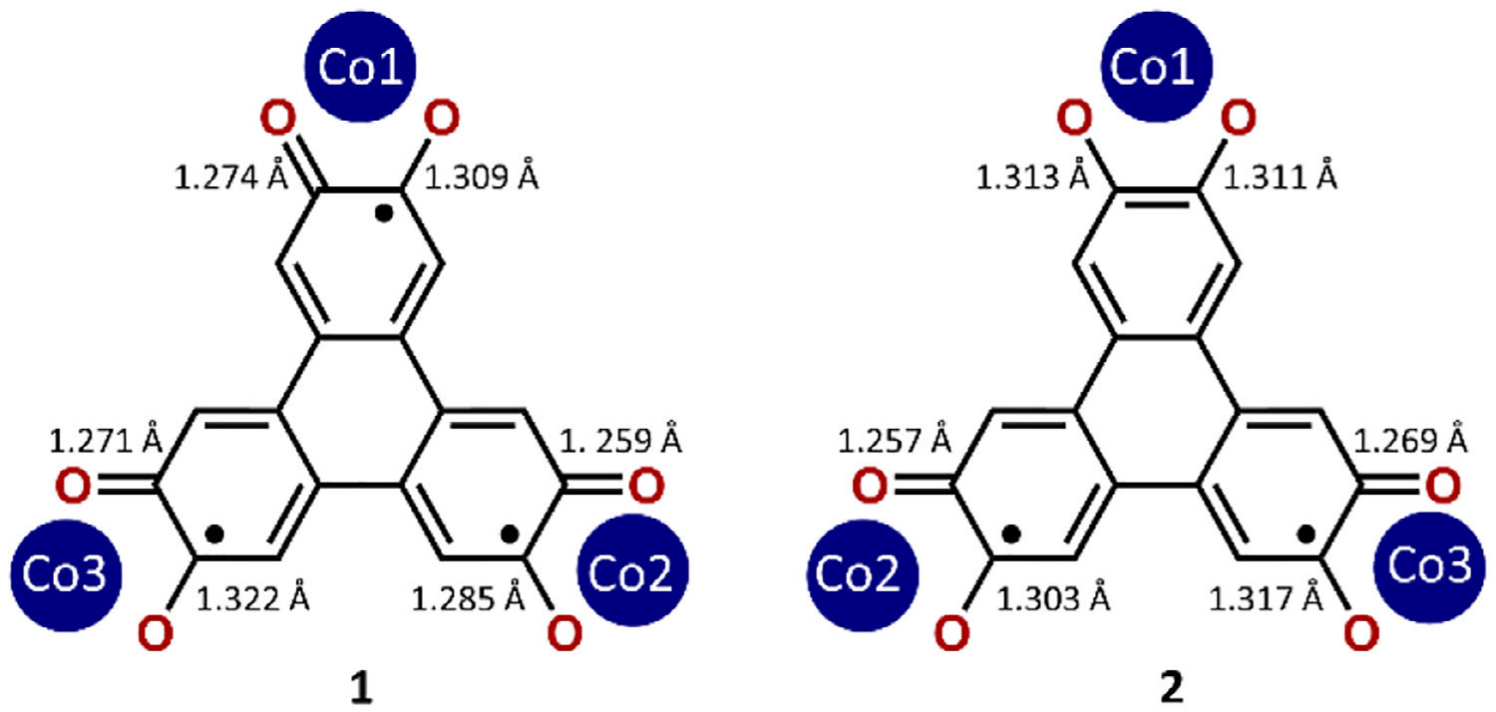
Bond distances and Co labeling in **1** and **2** displaying the semiquinone and catecholate OCCO groups.

Single‐crystal X‐ray Diffraction study reveals that **1** and **2** crystallize in the P2_1_/n and $P\bar{1}\left(2\right)$ space groups, respectively, with significant differences in cell parameters and volume cell (Table S2). For both compounds, the asymmetric unit comprises one molecule, perchlorate counter anions (3 for **1** and 2 for **2**), as well as solvent molecules. The Co atoms are all hexacoordinated to four N atoms of Me_3_TPA and two O atoms of HHTP (Figure 3, Figure S9). They adopt an octahedron geometry as confirmed by continuous shape measurements (Table S3).^[^ ^33^, ^34^ ^]^ The Co‐ligand bond lengths are in the range expected for Co(II) (Table S4), and χ*T* measurements performed in the 300–150 K range confirmed this oxidation state for both **1** and **2** (Figure S16). Therefore, using charge balance, HHTP is in the (sq‐sq‐sq)^3−^ state in **1** and the (cat‐sq‐sq)^4−^ state in **2**, demonstrating that the same pure species present in the solid state may be stabilized in solution. This assignment of the oxidation state is corroborated when examining the OCCO bond distances (Scheme 2). For complex **1,** the C‐O bond distances are all intermediate between typical single and double bonds, consistent with those found in semiquinones.^[^ ^4^, ^5^, ^17^ ^]^ The difference between the two C─O bond distances around Co1, Co2, and Co3 is 0.035 ± 0.012 Å, 0.026 ± 0.012 Å, and 0.050 ± 0.013 Å, respectively, suggesting that the semiquinone radicals are rather localized (on C1, C7, and C12, Scheme 2). This localized electronic distribution is similar to what was previously observed on similar complexes.^[^ ^4^, ^9^, ^17^ ^]^ For **2**, the C─O bond distances around Co1 are significantly longer (1.313(3) and 1.311(3) Å) and are identical, they correspond to the catecholate groups. On the other side of the molecule, the two radicals are also localized on C8 and C13, as evidenced by the large differences between the C─O bond distances around Co2 and Co3 (Δ = 0.046 ± 0.005 Å and 0.048 ± 0.006 Å, respectively). The localization of the radicals within the OCCO moieties impacts the Co‐O distances. They are shorter when involving the oxygen atom bearing the radical that is negatively charged, with values of 1.989 ± 0.010 Å in **1** and 1.986 ± 0.003 Å in **2,** while for the shorter C─O distances corresponding to the double bonds, they are longer (2.104 ± 0.009 Å for **1** and 2.088 ± 0.005 Å for **2**). Interestingly, in both complexes, the Me_3_TPA ligands are positioned so that the methyl groups are pointing toward the oxygen atom bearing the radical, as the increase of electronic density brought by CH_3_ strengthens the mesomeric forms depicted on Scheme 2.

The localization of the radicals onto the OCCO moieties for **1** confers to HHTP a particular electronic structure that was investigated by ab initio calculation on model complexes where Co(II) was replaced by Zn(II) (noted 1 _Zn_ and 2 _Zn_, see Supporting Information). The molecular orbitals (MOs) energy diagram shows the presence of three nondegenerate orbitals: one bonding and one antibonding MO localized on OCCO close to Co2 and Co3, and a nonbonding MO mainly localized close to Co1 (Figure S10). The coupling among the three semiquinone electrons (for **1**) leads to one ground doublet (D1, S = 1/2) well separated (ΔE > 4000 cm^−1^) from the excited doublet (D2) and quartet (Q, S = 3/2) (Table S5). The MOs energy diagram of 2 _Zn_ (Figure S11) and energy spectrum (Table S6) are consistent with the electronic structure deduced from the crystal structure of **2**, where the cat moiety is localized close to one of the Co moieties (Co1, Scheme 2). These results are consistent with the picture of HHTP in **1** bearing one unpaired electron mainly localized on the OCCO moiety close to one Co ion (Co1). Consequently, the first one‐electron reduction process is expected to be localized on one OCCO close to one Co, leading to a reduced species with no residual spin on HHTP, as deduced from the simple analysis of the crystal structure of **2** (see Supporting Information for comments on theoretical calculations).

**Figure 4:**
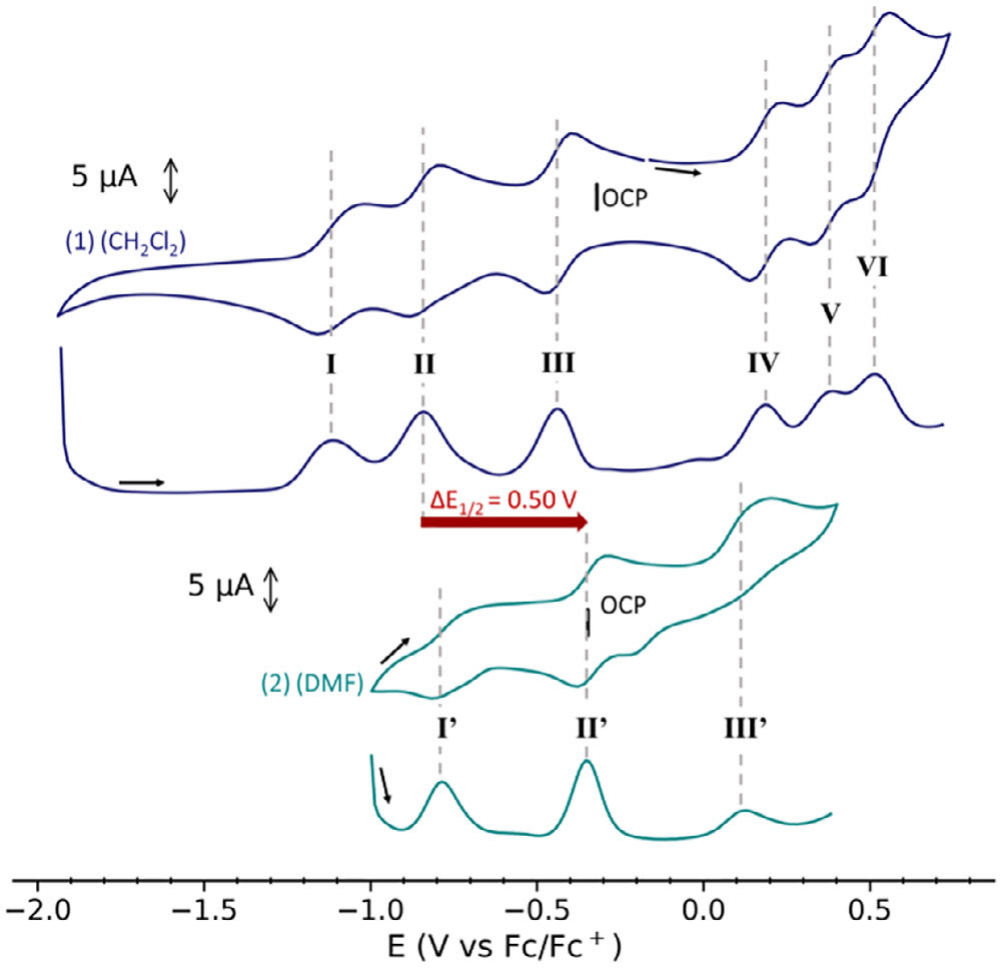
Cyclic voltammogram obtained at 100 mV/s and DPV of **1** in DCM and **2** in DMF (1 mM) using [NBu_4_]PF_6_ (0.1 M) as supporting electrolyte and Fc/Fc^+^ as internal reference.

Cyclic voltammetry (CV) measurements were performed on **1** in DCM and **2** in DMF (Figure 4, Figures S12–S14, and Table S7) and compared to that of [Co^III^_3_(TPA)_3_(HHTP)](BF_4_)_4_,^[^ ^18^ ^]^ (Figures S13, S15). All potentials are referred to vs the ferrocene/ferrocenium (Fc/Fc^+^) couple. The CV of **2** (Figure 4 and Figure S12), was recorded in DMF where the major species is (cat‐sq‐sq)^4−^ as found in the solid state. The observed three one‐electron redox processes (I′, II′, and III′, E_1/2_ = −0.75, −0.34 and +0.12 V), as shown by the differential pulse voltammogram (DPV), can be attributed to the sq^•−^/cat^2−^ couples based on the CV and the UV‐Vis‐NIR spectra of the parent complexes [M_3_(HBTp^(PhPh)^)_3_(HHTP)], (M = Co, Ni).^[^ ^9^ ^]^ The Open Circuit Potential (OCP) of **2** was recorded at −0.35 V and corresponds to the E_1/2_ of II′, which shows that the two species, (cat‐sq‐sq)^4−^ and (cat‐cat‐sq)^5−^, coexist in solution as already deduced from the electronic spectrum. The CV of **1** reveals six reversible and diffusion‐controlled redox processes (named I‐VI) (Figure 4 and Figures S13–S14), except for I and VI. The DPV is consistent with one‐electron events for all processes. The measured OCP value (−0.32 V), close to that of **2**, corresponds to the (sq‐sq‐sq)^3−^ state of HHTP, so the three reduction processes (*E*_1/2_ = −0.43, −0.84, and −1.11 V) are assigned to the three sq^•−^/cat^2−^ couples, respectively, since the Co(II) to Co(I) reduction is not possible. The three oxidation processes (*E*_1/2_ = +0.18, +0.37, and +0.52 V) can be HHTP‐centered but can also involve the oxidation of some of the Co(II) centers. Process VI is close to V and not completely reversible, likely due to compound adsorption on the electrode surface. Enlarging the potential acquisition window enables evidencing additional redox processes in oxidation that are not reversible due to the degradation of the complex (Figure S13b).

Therefore, the cyclovoltammetry study confirms that the solvent change from DMF to DCM enables stabilizing the (sq‐sq‐sq)^3−^ species by shifting the three redox processes by approx. −0.5 V.

**Scheme 3:**
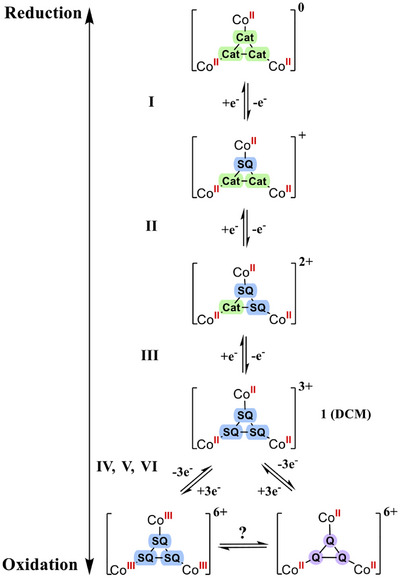
Proposed electronic pathways for the six redox processes observed for [Co_3_(Me_3_TPA)_3_(HHTP)]^3+^ in DCM.

The ancillary ligand Me_3_TPA not only stabilizes Co(II)‐sq over Co(III)‐cat but also considerably shifts the redox processes toward more negative potentials, enabling access to the oxidation processes that were not observed for [Co^III^_3_(TPA)_3_(HHTP)]^4+^ (Figure S13).^[^ ^18^ ^]^ Six redox processes have already been reported by the group of Ward on a diamagnetic Ru(II) trimetallic complex, but in their case the Δ*E*_1/2_ values for the oxidation and reduction events were very similar.^[^ ^35^ ^]^ In our case, the possible oxidation of Co(II) to Co(III) cannot be excluded, which might explain the decrease of Δ*E*_1/2_ values (from approx. 340 mV to 170 mV), comparable to that reported for the trinuclear Co_3_THT analogue (THT = triphenylene‐2,3,6,7,10,11‐hexathiolate).^[^ ^36^ ^]^ In addition, a Co(II)‐centered oxidation followed by an interconversion between Co(II)‐q and Co(III)‐sq species can occur, making it difficult to elucidate the phenomena involved in these oxidation processes, especially given that there are three reactive sites in the molecule. Such a process has already been suggested for bis(dioxolene) dinuclear cobalt complexes.^[^ ^27^, ^28^ ^]^ Also, the fact that three oxidation processes are reachable in **1** when only one oxidation process was observed for [Ni_3_(Me_3_TPA)_3_HHTP]^3+^,^[^ ^7^ ^]^ for [Ni_3_(BHTp^PhPh^)_3_(HHTP)] and for [Co_3_(BHTp^PhPh^)_3_(HHTP)] (at rather similar potentials)^[^ ^4^, ^9^ ^]^ highlights the role of the Co^II^(Me_3_TPA) moiety for accessing the three oxidation events. Scheme 3 gives a summary of the possible electronic pathways responsible for the redox waves observed in oxidation. In order to get a closer look into the electronic processes involved, we intend to perform spectro‐electrochemical measurements as well as further electrochemical experiments. The full investigation of the electronic processes requires the concomitant study of a family of related compounds and is underway.

## 3 Conclusion

In summary, this work presents an example of six‐electron reversible redox switch in a trimetallic Co(II) complex involving the redox‐active central bridging ligand HHTP. We demonstrated the ability to isolate the molecule in two different redox states, both in solution and in the solid state, which enabled characterizing in detailed electronic changes within the complexes. We showed that the modification of the ligand field around the Co(II) centers enables accessing additional oxidation events compared to parent complexes. Future work will focus on elucidating the nature of the electronic events, especially in oxidation, that may involve both the redox‐active HHTP ligand and also the Co^II^/Co^III^ redox couples.

## Supporting Information

CCDC 2350894 & 2350895 contains the supplementary crystallographic data for this paper. These data can be obtained free of charge from the Cambridge Crystallographic Data Centre and Fachinformationszentrum Karlsruhe via http://www.ccdc.cam.ac.uk/structures/. Supporting Information: Materials and methods, UV‐Vis‐NIR spectroscopy and TD‐DFT, Infrared spectroscopy, Single‐Crystal X‐ray Analysis, Theoretical calculations, Electrochemistry. The authors have cited additional references within the Supporting Information.^[^ ^37^, ^38^, ^39^, ^40^, ^41^, ^42^, ^43^, ^44^, ^45^, ^46^, ^47^, ^48^, ^49^, ^50^, ^51^, ^52^, ^53^ ^]^

## Conflict of Interest

The authors declare no conflict of interest.

## Supporting information



Supporting Information

## Data Availability

The data that support the findings of this study are available from the corresponding author upon reasonable request.
