# Supplementary material for: Solvatochromism and Redox Multi‐Switch in a Trinuclear Cobalt(II) Complex
Source: Chemistry. 2025 Jul 7;31(45):e202501455. doi: 10.1002/chem.202501455 (PMC12351425; doi:10.1002/chem.202501455)
Supplement: Supplementary file 1 — Supporting Information [file CHEM-31-e202501455-s001.docx]

**Supporting Information**

Solvatochromism and Redox Multi-Switch in a Trinuclear Cobalt(II) Complex

Solène Delaporte,^[a]^ Nathalie Bridonneau,^[a]^ François Lambert,^[a]^ Régis Guillot,^[a]^ Nicolas Suaud,^[b]^ Nathalie Guihéry,^[b]^ Ganping Wang,^[c]^ Thayalan Rajeshkumar,^[c]^ Laurent Maron,^[c]^ Talal Mallah^[a]^

nathalie.bridonneau@universite-paris-saclay.fr

[a] S. Delaporte, Dr. F. Lambert, Dr. N. Bridonneau, Dr. R. Guillot, Pr. T. Mallah
Institut de Chimie Moléculaire et des Matériaux d’Orsay
CNRS, Université Paris-Saclay UMR 8182
17, avenue des Sciences, 91400, Orsay (France)

E-mail: nathalie.bridonneau@universite-paris-saclay.fr

[b] Dr. N. Suaud, Pr. N. Guihéry

Laboratoire de Chimie et Physique Quantiques (LCPQ),

Université de Toulouse, CNRS,

118 route de Narbonne,

F-31062 Toulouse (France)

[c] G. Wang, Dr. T. Rajeshkumar, Pr. L. Maron
Université de Toulouse, INSA, CNRS
135, avenue de Rangueil
F-31077 Toulouse (Fr)

Contents

[S1. Materials and methods 2](#_Toc196834386)

[General 2](#_Toc196834387)

[Instrumentation 2](#_Toc196834388)

[Synthesis 3](#_Toc196834389)

[Mass spectrometry 5](#_Toc196834390)

[S2. UV-Vis NIR spectroscopy and TD-DFT 7](#_Toc196834391)

[Experimental 7](#_Toc196834392)

[TD-DFT 7](#_Toc196834393)

[S3. Infrared spectroscopy 14](#_Toc196834394)

[S4. Single-Crystal X-ray Analysis 16](#_Toc196834395)

[S5. Theoretical calculations 19](#_Toc196834396)

[S6. Electrochemistry 21](#_Toc196834397)

[S7. Magnetic characterizations 24](#_Toc196834398)

[References 25](#_Toc196834399)

## S1. Materials and methods

### General

All the reagents used for these syntheses were purchased commercially and were used directly without further purification unless mentioned. All syntheses reported here were carried out under aerobic conditions using non-anhydrous solvent unless mentioned. Syntheses of Me_3_TPA and its precursors were conducted following reported procedures with slight modifications^[1,2]^. The synthesis of [Co_3_(TPA)_3_HHTP](BF_4_)_4_ was adapted from the work of Y. Suenaga and co-workers,^[3]^ and used as a model for comparisons.

***Caution:*** *Perchlorate salts are highly reactive and can be explosive under certain conditions; handle with extreme care and follow all safety protocols.*

### Instrumentation

#### FT-IR

FT-IR spectra have been obtained on a Perkin Elmer Spectrum 100 FT-IR Spectrometer in the 450-4000 cm^-1^ using KBr pellets. Abbreviations: v (very), w (weak), m (medium), s (strong), br (broad).

#### UV-Vis-NIR spectroscopy

UV-Visible-NIR spectra have been obtained using a Cary 5000 UV-Vis-NIR Spectrometer from Varian in double beam mode in Quartz cuvettes.

#### Electrochemistry

Cyclic-voltammogram and differential pulse voltammogram measurements were carried out in anhydrous solvent with 0.1M TBAPF_6_ as a supporting electrolyte, and recorded using an AUTOLAB PGSTAT320, a glassy carbon working electrode, a platinum wire counter electrode, and an Ag/AgCl reference electrode. Solvent was degassed with argon flux prior any measurements. Ferrocene was added at the end of each experiment as an intern reference.

**^1^H NMR spectra** were recorded on a Bruker AV-300 or Bruker AV-400.

**Mass spectra** were recorded on a Bruker MicroTOF-Q spectrometer by electrospray ionization (ESI).

**Elemental analysis (C, H, N)** was performed by the chromatography platform UMR 8076 BioCIS - Université Paris-Saclay, on a Perkin Elmer CHN 2400.

**Theoretical Calculations**

*Ab initio*. Calculations were performed on both complexes where the magnetic Co(II) ions were replaced by diamagnetic Zn(II) ones in order to investigate the spin state of the HHTP ligand in its -4 and -3 oxidation states. Since **2** presents an H-bond between a H_2_O molecule and an O atom, H_2_O and ClO_4_^-^ to which it makes another H-bond are explicitly considered in the calculations. A first step consists in the optimization of the position of H atoms (while the position of all other atoms is taken from X-Ray data) using DFT (PBE functional, D3 dispersion correction) and def2-SVP atomic basis sets (5s3p2d1f fo Zn, 3s2p1d for C, N and O, 4s3p1d for Cl and 2p1p for H). CASSCF+NEVPT2 calculations were then performed using Def2-TZVP atomic basis sets for C and O (5s3p2d1f) atoms of the HHTP ligand and de2-SVP for other atoms. For **1** a CAS(3/3), *i.e.* 3 active electrons in 3 active MO, is considered while CAS(4/3) were performed for **2**.

*Time-Dependent DFT*. All the DFT calculations were performed using Gaussian09 suite of programs.^[4]^ Hybrid DFT functional (B3PW91)^[5,6]^ along with Relativistic Effective Core Potentials with associated basis sets were used to describe for Co atoms^[7,8]^ and 6-31G** basis sets for rest of the atoms.^[9,10]^ To account for the solvation effects, SMD model using Dichloromethane solvent has been included in the geometry optimization.^[11]^ Geometry optimization were carried out without any symmetry constraints and the nature of the optimized structures (minima) were confirmed by frequency calculations. UV-Visible spectra were simulated using Time Dependent DFT approaches where solvent effects were considered using the SMD model using either dichloromethane (DCM) or DimethylFormamide (DMF) as solvent.

### Synthesis

#### 2,3,6,7,10,11-hexahydroxytriphenylene (H_6_HHTP)

Veratrole (6.91 g, 50 mmol) was dissolved in 50 mL CH_2_Cl_2_ and transferred to a dropping funnel. In a round-bottom flask, FeCl_3_ (25.5 g, 157.2 mmol) was dissolved in 150 mL CH_2_Cl_2_. H_2_SO_4_ 95% (0.350 mL) was added to the Fe suspension, and the veratrole solution was added dropwise to the mixture under stirring. The reaction was left to proceed for 3h at room temperature, resulting in a black solution. MeOH (200 mL) was added slowly to give an orange mixture. Stirring was continued for an additional 30 minutes. The product was filtered, washed with MeOH (3 x 50 mL), and dried at 45°C. A white solid of hexamethoxytriphenylene (HMTP) was obtained. (5.7 g, 82%). HMTP (2.5 g, 6.1 mmol) was then added to a round flask. Glacial acetic acid (90 mL) and HBr (90 mL) were added. The mixture was placed under argon and heated under reflux for 24h. The white solution turned blue, and upon cooling to room temperature, a white solid precipitated. The solid was washed with cold water and dried. (1.9 g, 95 %). **^1^H NMR** (300 MHz, DMSO-d_6_) δ 9.26 (s, 6H), 7.60 (s, 6H).

#### Tris(6-methyl-2-pyridylmethyl)amine (Me_3_TPA)

6-methyl-2-pyridinecarboxaldehyde (2 g, 16.5 mmol) and hydroxylamine hydrochloride (1.38 g, 19.8 mmol) were dissolved in EtOH (6 ml) at room temperature. K_2_CO_3_ (2.74 g, 19.8 mmol) in 12mL H_2_O was then added slowly. After refluxing for 1h, the mixture was cooled in an ice bath, and the resulting white solid was collected by filtration. The crude oxime (1,8 g, 13.2 mmol) was used without further purification and dissolved in 80% aqueous acetic acid (20 mL). Zinc powder (3.8 g, 58.2 mmol) was added portion-wise over an ice bath. The reaction mixture was then refluxed for 2h and stirred at room temperature overnight. TLC on alumina using DCM/MeOH (9:1) confirmed the completion of the reaction. After filtration through Celite, solid NaOH was added to the filtrate until pH = 12-14. The mixture was filtered through Celite again to remove the white precipitate. The filtrate was then extracted with DCM/MeOH 5% (3 x 100 mL), and the organic phase was evaporated under reduced pressure to afford a colorless oil that quickly turned yellow. The exact amount of amine obtained was determined with ^1^H NMR (0.51 g, 31%) **^1^H NMR** (300 MHz, Chloroform-*d*) δ 7.54 (t, 1H), 7.05 (dd, 2H), 3.95 (s, 2H), 2.55 (s, 3H). Subsequently, 6-methyl-pyridine-2-carboxyaldehyde (1.0 g, 8.3 mmol) was added to a stirred mixture of 6-methyl-2-aminomethylpyridine (0.51 g, 4.15 mmol) and sodium triacetoxyborohydride (3.52 g, 16.6 mmol) in dry dichloromethane (40 mL). The resulting mixture was stirred for 18 h, after which a saturated aqueous solution of sodium hydrogenocarbonate (30 mL) was added. The reaction mixture was stirred until effervescence ceased and then extracted with ethyl acetate (3x20 mL). The organic fraction was separated, dried over MgSO_4_, and the solvent was removed under reduced pressure. The residue was extracted several times with hot pentane, and the solvent was removed to give a light-yellow solid. (1.25 g, 91 %) **^1^H NMR** (400 MHz, Chloroform-*d*) δ 7.54 (t, 3H), 7.43 (d, 3H), 6.99 (d, 3H), 3.88 (s, 6H), 2.51 (s, 9H).

#### [Co_3_(Me_3_TPA)_3_HHTP](ClO_4_)_n_ (n = 2 and 3, crude compound)

Me_3_TPA (100 mg, 0.3 mmol) in 5 mL MeOH was added to a solution of Co(ClO_4_)_2_.6H_2_O (110 mg, 0.3 mmol) in 5 mL MeOH under stirring. The pink cobalt solution turned dark pink and was left under stirring for 10 minutes. H_6_HHTP (32 mg, 0.1 mmol) was deprotonated with Et_3_N (84 µL, 0.6 mmol) in 30 mL MeOH, and was slowly added to the previous solution that turned brown, and blue after a few seconds. After 1h of stirring, the solution was filtrated, and a dark blue powder was isolated by Et_2_O precipitation and filtration. **FT-IR (KBr)** ν/cm^-1^ 3436 (s, br), 3072 (w), 3015 (w), 2924 (w), 1606 (m), 1578 (w), 1515 (s), 1471 (m), 1453 (m), 1368 (w), 1308 (m), 1268 (w), 1243 (w), 1225 (m), 1167 (w), 1096 (vs, ClO_4_^-^), 1010 (m), 972 (w), 917 (w), 889 (w), 863 (w), 790 (m), 623 (m), 538 (m).  **ESI/MS (CH_3_CN) m/z** 745.70 [M]^2+^, 497.47 [M]^3+^.

#### [Co_3_(Me_3_TPA)_3_HHTP](ClO_4_)_3_ (1)

*Method 1*. Crude powder of [Co_3_(Me_3_TPA)_3_HHTP](ClO_4_)_2-3_ was dissolved in a minimum volume of dichloromethane. Toluene was layered onto the complex solution and afforded black needles of **1** in small yield. *Method 2*. Me_3_TPA (100 mg, 0.3 mmol) in 5 mL DCM was added to Co(ClO_4_)_2_.6H_2_O (110 mg, 0.3 mmol) in 5 mL DCM under vigorous stirring until turning brown. H_6_HHTP (32 mg, 0.1 mmol) was deprotonated with Et_3_N (84 µL, 0.6 mmol) in 30 mL DCM, and was slowly added to the previous solution. The dark solution slowly turned dark blue-purple after 1h under vigorous stirring. The solution was filtrated, and a dark blue powder was isolated by Et_2_O precipitation and filtration. Crystals of **1** were obtained in quantitative yield by layering toluene on a DCM solution of the isolated powder. **FT-IR (KBr) ν/cm^-1^** 3427 (s, br), 3065 (w), 3020 (w), 2923 (w), 1606 (m), 1578 (w), 1518 (vs), 1453 (s), 1385 (w), 1369 (w), 1353 (w), 1328 (m), 1312 (w), 1272 (w), 1240 (m), 1227 (m), 1166 (w), 1145 (m), 1091 (vs, ClO_4_^-^), 1010 (m), 970 (w), 917 (w), 890 (w), 790 (m), 624 (m), 536 (m). **ESI/MS (CH_3_CN)** **m/z** 745.70 [M]^2+^, 497.47 [M]^3+^. Analyzed as [Co_3_(Me_3_TPA)_3_(HHTP)](ClO4)_3_‧2H_2_O Anal. calcd for C81H82N12Cl3Co3O20: C, 53.26; H, 4.52; N, 9.20 %. Found: C, 53.24; H, 4.37; N, 9.11%.

#### [Co_3_(Me_3_TPA)_3_HHTP](ClO_4_)_2_ (2)

Characterization in solution were conducted by dissolving a crude powder of [Co_3_(Me_3_TPA)_3_HHTP](ClO_4_)_2-3_ in DMF. Slow diffusion of diethyl ether into a concentrated DMF solution afforded dark blue crystals of **2** suitable for SXRD in small yield. **FT-IR (KBr)** ν/cm^-1^ 3428 (s, br), 3069 (w), 3011 (w), 2923 (w), 1660 (m, DMF), 1605 (m), 1578 (w), 1514 (s), 1472 (s), 1452 (s), 1389 (w), 1368 (w), 1308 (s), 1266 (w), 1224 (m), 1164 (w), 1145 (w), 1093 (s, ClO_4_^-^), 1009 (w), 971 (w), 942 (w), 916 (w), 890 (w), 864 (w), 790 (m), 626 (m), 543 (m).

#### [Co_3_(TPA)_3_HHTP](BF_4_)_4_ (3)

TPA (87 mg, 0.3 mmol) in 10 mL MeOH was added to a solution of Co(BF_4_)_2_.6H_2_O (102 mg, 0.3 mmol) in 10 mL MeOH under stirring. The purple cobalt solution turned brown and was left under stirring for 10 minutes. H_6_HHTP (32.5 mg, 0.1 mmol) was deprotonated with Et_3_N (84 µL, 0.6 mmol) in 30 mL MeOH and was slowly added to the previous solution that immediately turned dark green, with the formation of a green precipitate. After 20 minutes of stirring, a dark green solid was collected by filtration and washed with a minimum amount of methanol. **FT-IR (KBr)** ν/cm^-1^ 3444 (s, br), 3072 (w), 2939 (w), 1636 (w, br), 1610 (m), 1574 (w), 1480 (s), 1434 (s), 1377 (w), 1260 (s), 1084 (s, BF_4_^-^), 992 (w), 890 (w), 817 (m), 772 (m), 732 (w), 665 (w), 616 (w), 533 (w), 522 (w). **ESI/MS (CH_3_CN) m/z** 455.09 [M]^3+^.

### Mass spectrometry

The presence of the complexes in solution was probed using positive ESI mass spectrometry in acetonitrile. Mass spectrometry on solution of the crude Me_3_TPA complex, **1** and **3** demonstrated the presence of the compounds. Experimental and simulated^[12]^ spectra are respectively displayed in purple and blue on **Figure S1**.

| 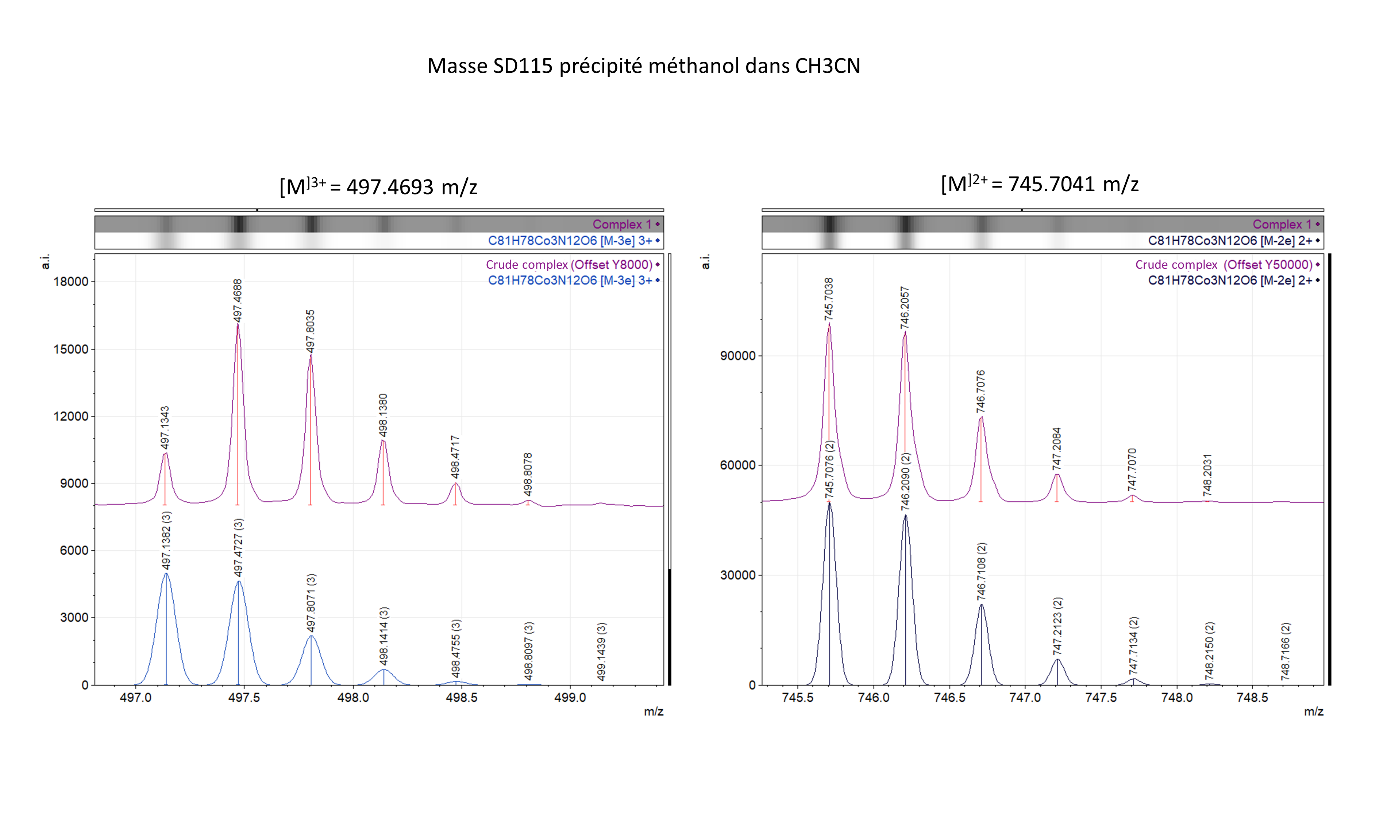 |
| --- |
|  |
| 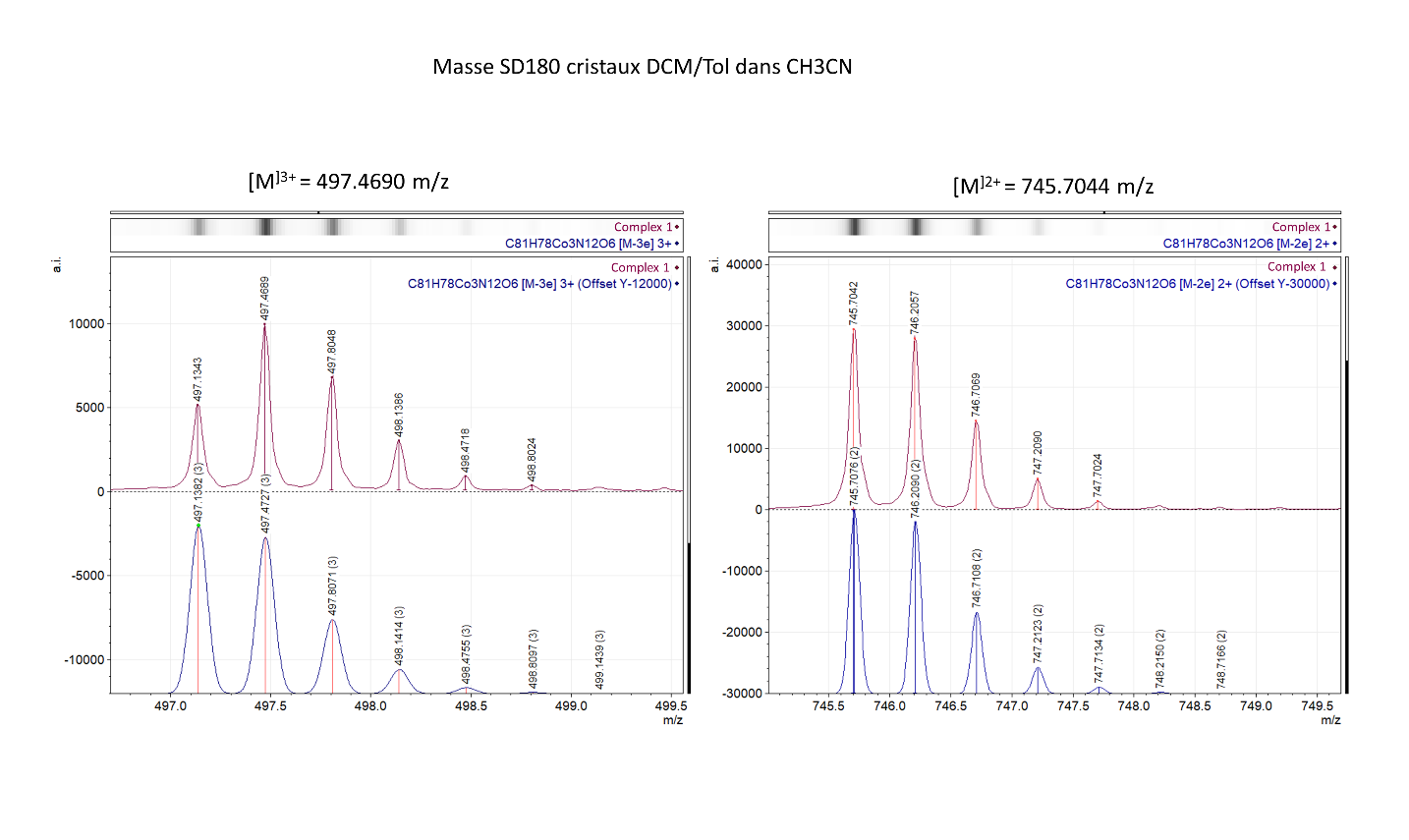 |
| (b) |
| 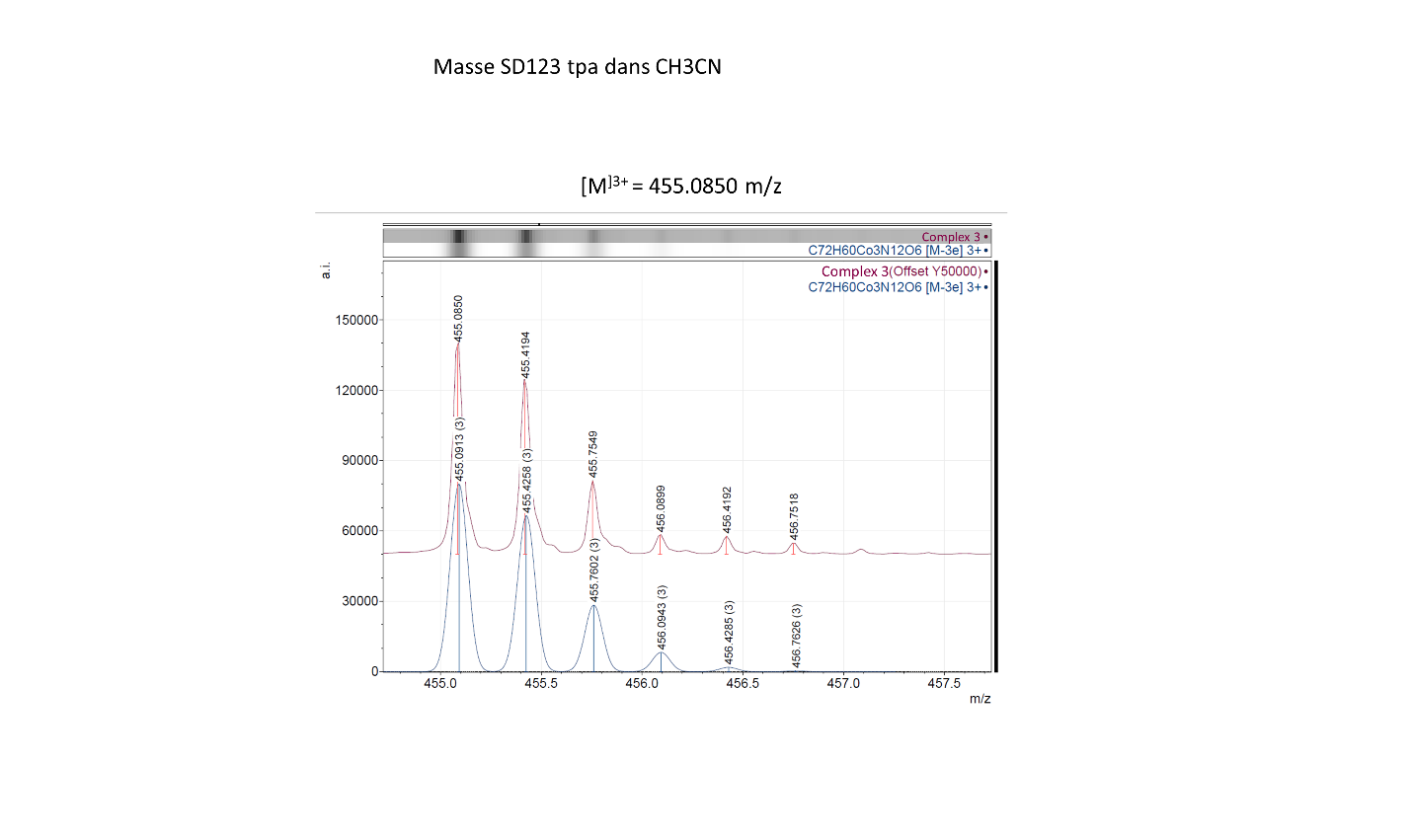  (c) |
| **Figure S1**. Experimental (top, purple) and simulated^[12]^ (bottom, blue) mass spectra of (a) crude complex, (b) **1** and (c) **3** in CH_3_CN. |

## S2. UV-Vis NIR spectroscopy and TD-DFT

### Experimental

| 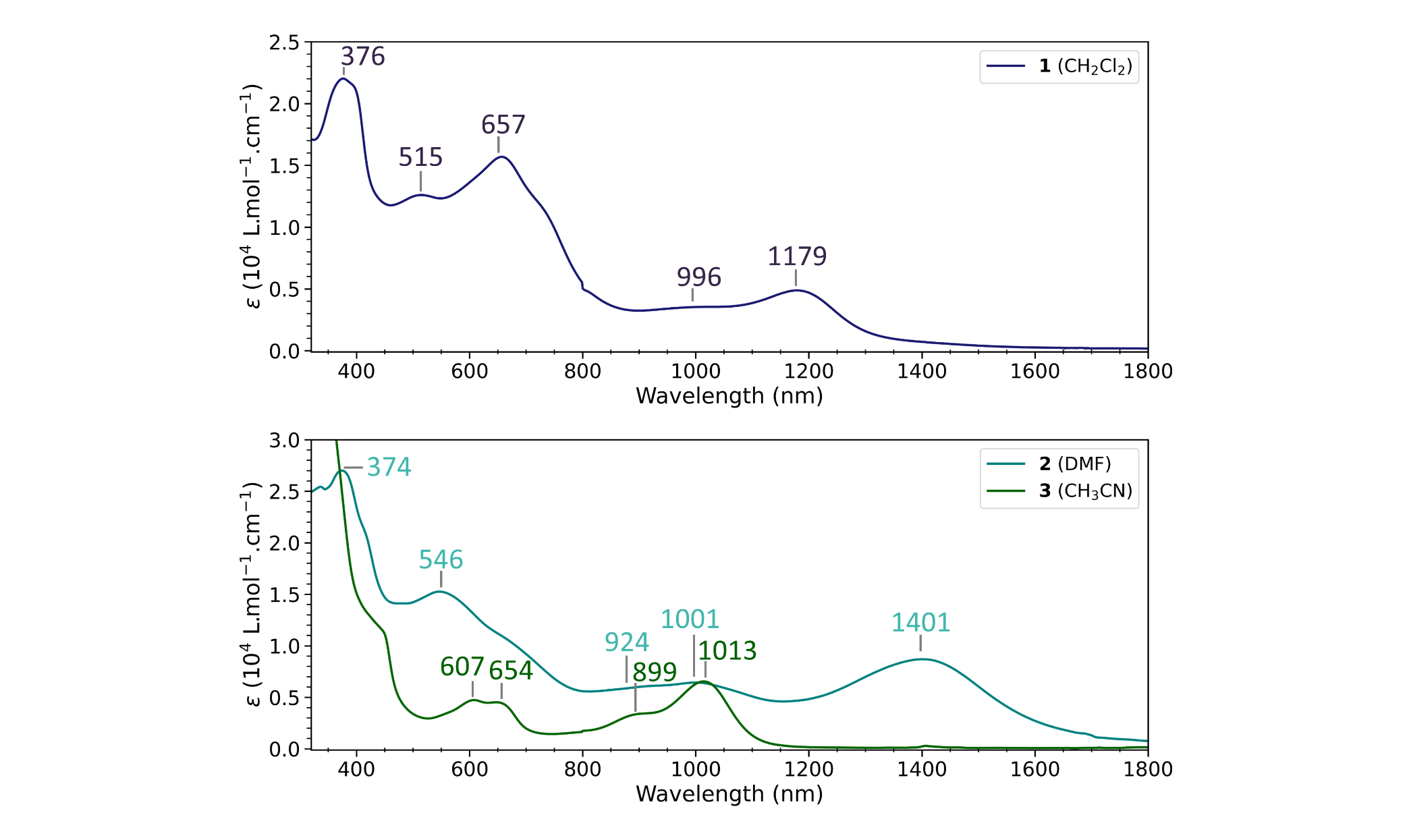 |
| --- |
| **Figure S2.** UV-Vis NIR absorption spectra of **2** in DMF and **3** in CH_3_CN (bottom). |

As mentioned in the main text, the OCP of **2** in DMF is relatively close the half-potential of the redox-process II’. In addition, its electronic spectrum displays two weak absorption bands at 924 and 1001 nm, that were not always observed. Comparing this spectrum with the one of **3** in CH_3_CN suggests the presence of a minority [Co^II^_3_(cat-cat-sq)]^+^ state in equilibrium with the identified [Co^II^_3_(cat-sq-sq)]^2+^.

### TD-DFT

In this study, a systematic investigation of the complexes has been conducted computationally in order to minimize the potential errors. Therefore, although not reported in the present communication, the investigation started from the neutral triscatecholate complex of cobalt that was then oxidized to a biscatecholate-semi-quinone cationic complex, that has been further oxidized and so on. In all cases, the geometry of the complex has been fully optimized in solution and two different spin states were computed.


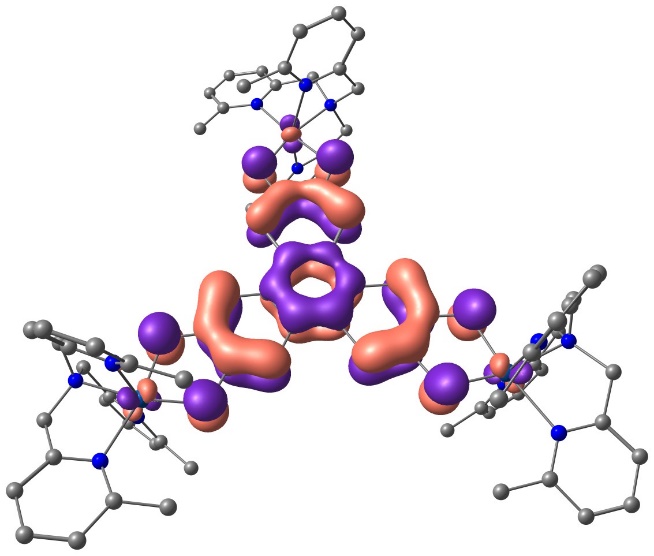

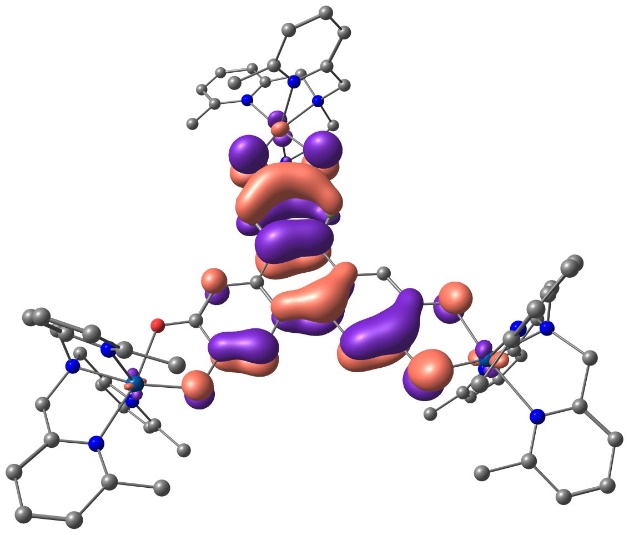


SOMO1 SOMO2


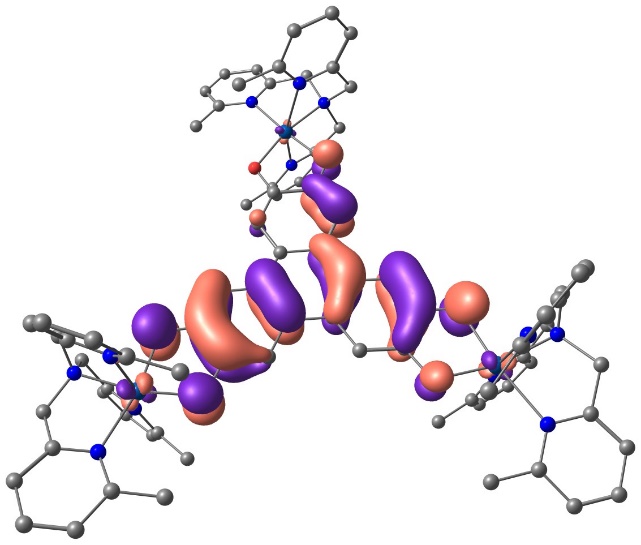

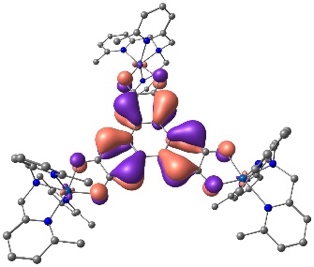


SOMO3 HOMO

Figure S3. Three Singly Occupied (SOMO) and Highest Occupied (HOMO) orbitals of the (sq_sq_sq)^3-^ in complex **1**


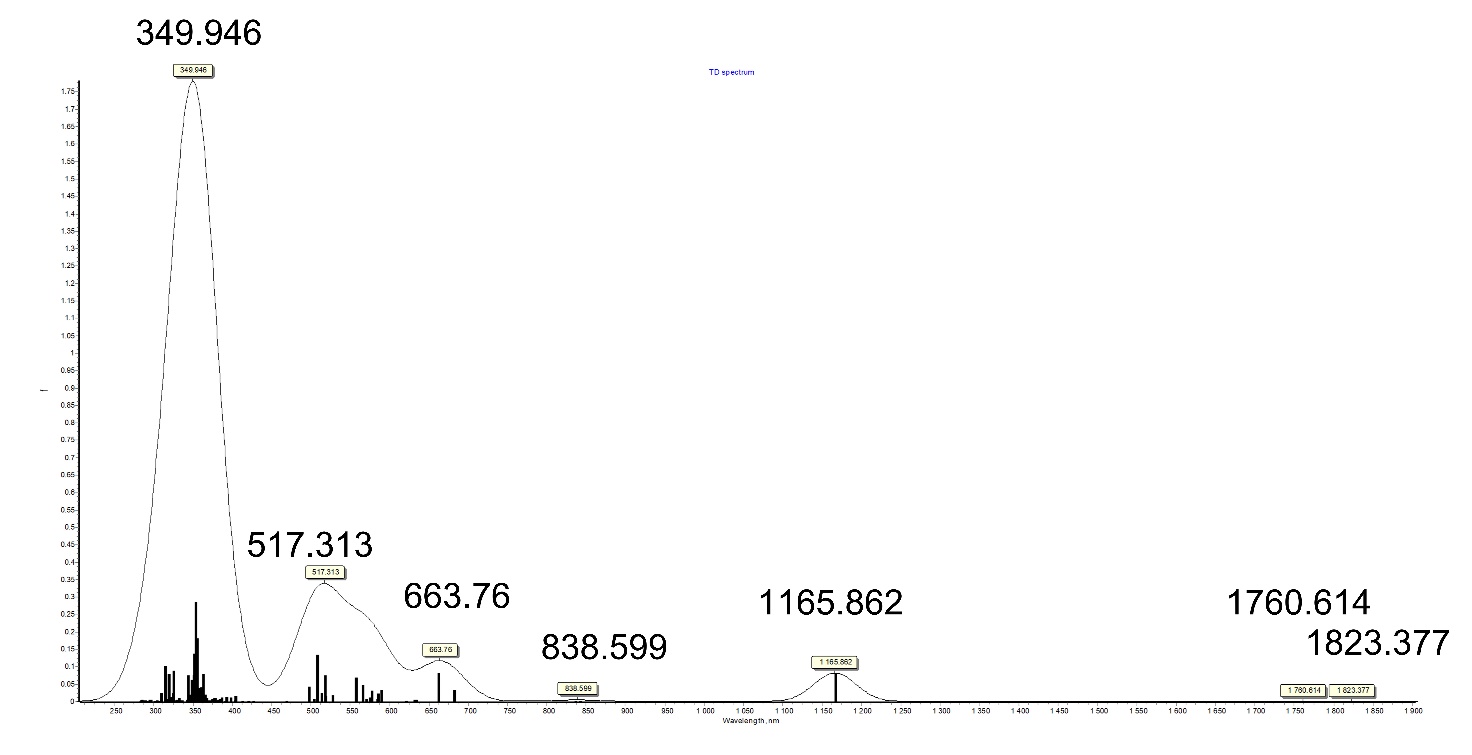


Figure S4. TD-DFT spectrum of complex **1** in DCM.


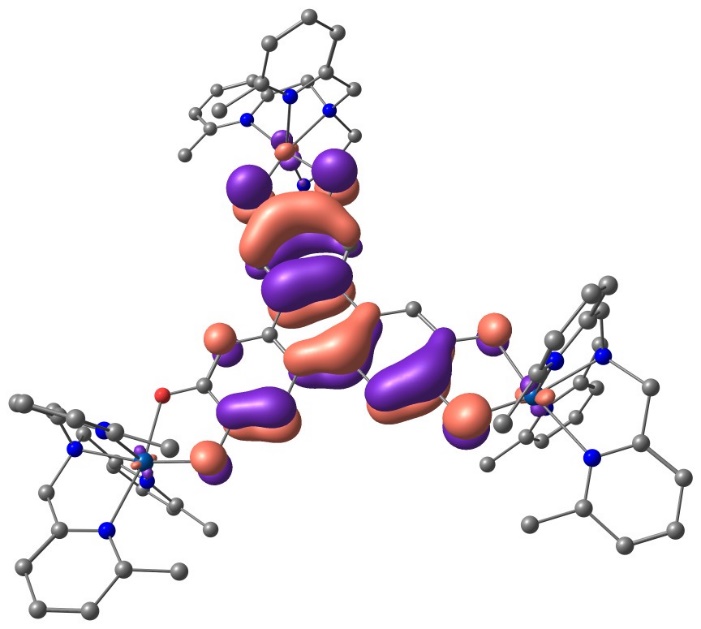

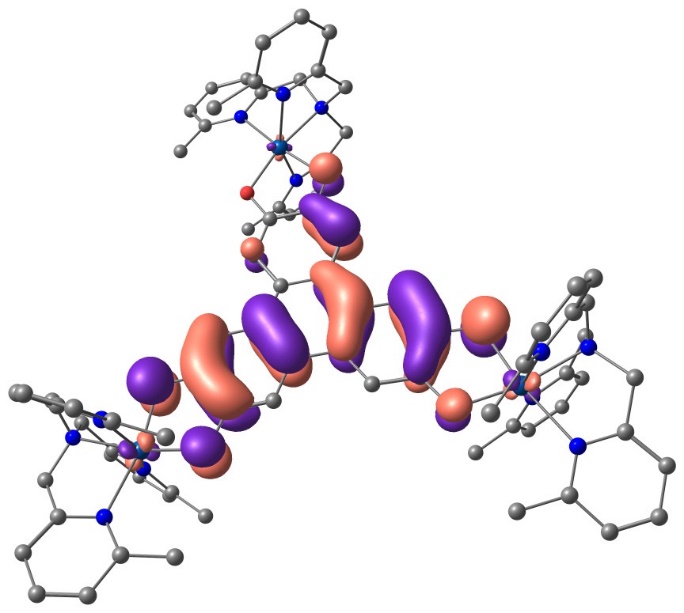


Figure S5. Singly Occupied (SOMO) orbitals of the (sq_sq_cat)^4-^ in complex **2**


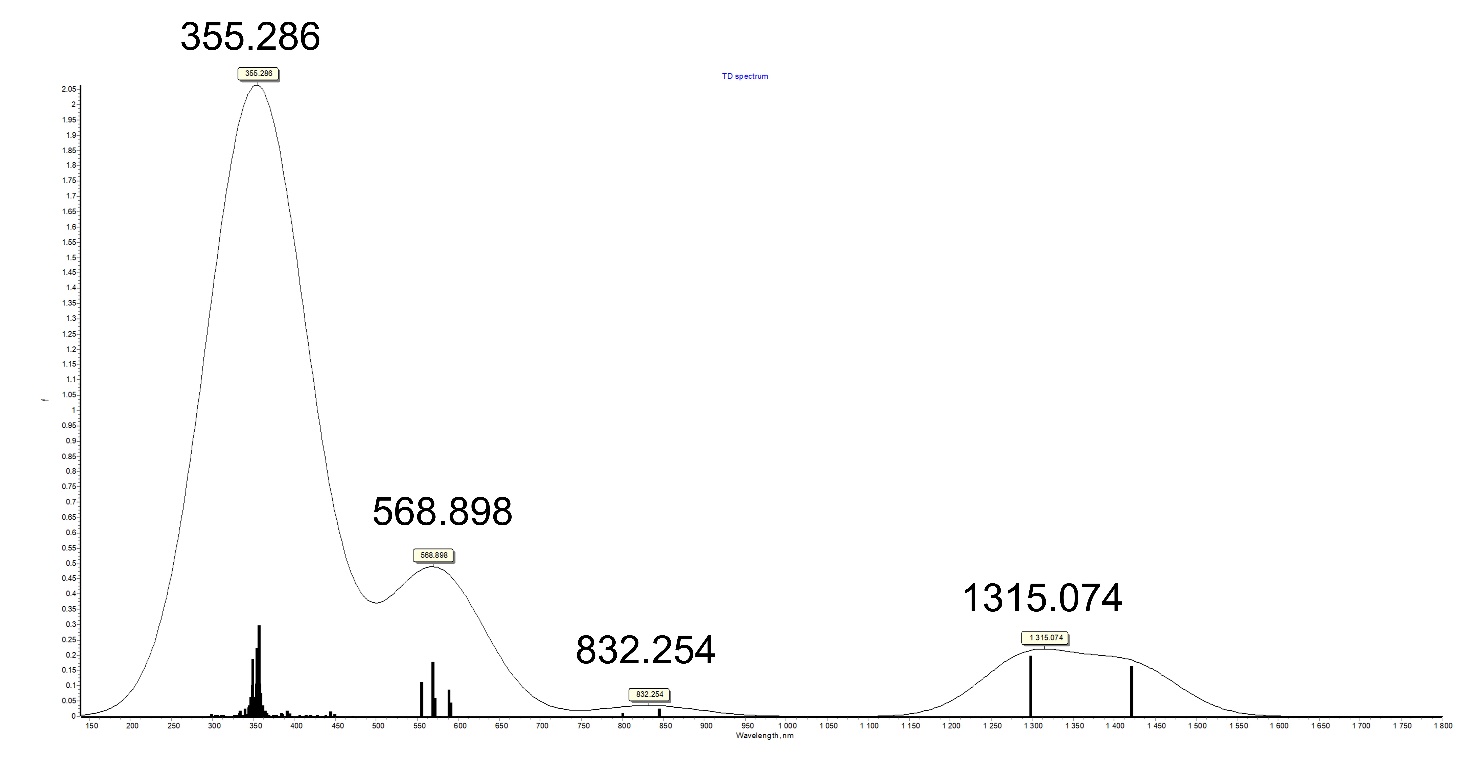


Figure S6. TD-DFT spectrum of complex **2** in DMF

Figure S7. Representation of the two excitations responsible of the transition observed in the 1400 nm region for complex **2**. The transition around 550 nm is due to an excitation from the HOMO-1 to the SOMOs and is a LLCT band.

Cartesian coordinates of the optimized structures

| Complex **1**-HS | Complex **2**-HS |
| --- | --- |
| Co 8.090248 18.189017 22.867756  Co 12.194301 7.725207 22.391852  Co 12.294335 14.201292 13.283440  O 8.747574 17.724987 20.963592  O 8.688077 16.174823 23.115477  O 11.351240 9.610235 22.828441  O 12.351258 8.683211 20.556730  O 12.374926 12.772826 14.774282  O 11.546415 15.289692 14.937299  N 13.114243 5.733168 22.332635  N 14.208789 15.300492 13.534637  N 7.600066 18.310663 24.962247  N 5.984750 17.468684 22.925181  N 7.582722 20.324980 22.969112  N 10.116492 18.894308 23.775724  N 11.594246 7.033853 24.342819  N 10.115722 6.855776 22.030691  N 13.967846 8.354028 23.617162  N 12.764658 13.306276 11.333407  N 10.104264 13.895430 12.569180  C 11.330886 10.390095 21.814490  C 10.272559 13.882660 20.776187  C 11.368944 19.360213 21.714498  H 11.925051 20.220638 21.333419  H 10.412158 19.282022 21.199720  H 11.947039 18.461036 21.466601  C 7.750050 21.282185 22.031931  C 7.636066 22.638429 22.357303  H 7.775795 23.381674 21.578598  C 7.354915 23.013708 23.662212  H 7.278659 24.063030 23.932560  C 7.167173 22.020510 24.619095  H 6.932932 22.265918 25.650153  C 7.273992 20.689798 24.231641  C 8.037661 20.887016 20.615054  H 8.927208 21.408062 20.246109  H 7.204496 21.187490 19.969036  H 8.189517 19.813850 20.506753  C 6.926897 19.599815 25.214783  H 7.114437 19.942511 26.239544  H 5.845805 19.437735 25.133766  C 8.852867 18.169953 25.725442  H 9.122078 17.108325 25.717451  H 8.711629 18.467071 26.772558  C 10.003678 18.930225 25.119064  C 11.196698 19.458458 23.199237  C 12.177119 20.099218 23.967616  H 13.029750 20.548656 23.468262  C 12.046321 20.155572 25.347111  H 12.793530 20.658103 25.954754  C 10.937649 19.555878 25.937675  H 10.792410 19.568471 27.013110  C 6.691366 17.179167 25.229378  H 6.226822 17.261008 26.220538  H 7.289320 16.262728 25.215069  C 5.631762 17.075350 24.163106  C 4.369351 16.567387 24.453742  H 4.123425 16.273393 25.469111  C 3.446400 16.450314 23.419563  H 2.453628 16.051683 23.607936  C 3.808485 16.867547 22.144736  H 3.108445 16.806849 21.317717  C 5.086830 17.387170 21.921818  C 9.225561 16.534642 20.859324  C 9.199178 15.685938 22.049780  C 9.726456 14.379630 21.955241  H 9.678459 13.779908 22.856299  C 10.297961 14.727861 19.598205  C 9.771914 16.016025 19.671797  H 9.764101 16.676409 18.812733  C 5.481463 17.903925 20.573096  H 4.781773 17.562586 19.806645  H 6.491949 17.586417 20.306267  H 5.471065 19.000418 20.573465  C 10.816768 12.522353 20.714261  C 11.362711 12.014796 19.470012  C 11.378618 12.865711 18.276142  C 10.868547 14.221111 18.346840  C 10.819777 11.704804 21.839869  H 10.432680 12.047644 22.791962  C 11.882826 9.882445 20.560662  C 11.877634 10.720839 19.430294  H 12.302447 10.305492 18.524358  C 11.886533 12.391126 17.068203  H 12.264460 11.380690 16.968874  C 11.934385 13.189052 15.910079  C 11.456396 14.567952 15.988894  C 10.926876 15.027015 17.214802  H 10.572229 16.050475 17.226263  C 10.141678 7.286737 24.426023  H 9.998748 8.368205 24.512906  H 9.704325 6.818046 25.317233  C 9.426788 6.809667 23.187317  C 9.518480 6.442826 20.893162  C 8.182725 6.027341 20.891639  H 7.725229 5.716492 19.957776  C 7.466571 6.000642 22.081158  H 6.431088 5.672821 22.095333  C 8.105038 6.379920 23.257231  H 7.593748 6.350758 24.213976  N 12.019120 15.938546 12.038481  C 12.715452 17.040371 12.729453  H 12.109822 17.324401 13.595085  H 12.812653 17.923427 12.084635  C 14.071241 16.602354 13.222499  C 15.397927 14.845954 13.982247  C 16.471732 15.719816 14.174259  H 17.414006 15.330618 14.546228  C 16.324942 17.068434 13.873006  H 17.152971 17.757738 14.011370  C 15.107975 17.518519 13.372108  H 14.956568 18.558254 13.099373  C 12.963903 5.096045 23.512048  C 13.691687 3.957689 23.841649  H 13.548826 3.484954 24.808225  C 14.592094 3.446400 22.911961  H 15.178817 2.561339 23.140984  C 14.725986 4.086168 21.688218  H 15.411191 3.712616 20.933831  C 13.974027 5.234499 21.418945  C 12.324646 7.838611 25.337720  H 12.304956 7.360985 26.325115  H 11.805394 8.798367 25.430626  C 13.743793 8.137512 24.929648  C 14.737816 8.275946 25.890964  H 14.513100 8.081225 26.934767  C 16.008406 8.676351 25.484695  H 16.807879 8.796664 26.210232  C 16.233561 8.917527 24.138136  H 17.208383 9.235375 23.781369  C 15.194519 8.740591 23.215874  C 11.890272 5.590446 24.450526  H 10.977087 5.037306 24.203201  H 12.144437 5.323868 25.482944  C 10.330955 6.381563 19.637353  H 9.685393 6.398427 18.755660  H 10.898428 5.443315 19.611086  H 11.044551 7.205459 19.583612  C 12.646469 14.210934 10.338472  C 12.641647 13.847108 8.997314  H 12.527933 14.607291 8.230999  C 12.793749 12.502922 8.667667  H 12.792980 12.186016 7.628748  C 12.951938 11.578185 9.688671  H 13.086898 10.522825 9.473355  C 12.931929 12.004086 11.021621  C 12.628474 15.669332 10.721740  H 13.673165 15.998748 10.760535  H 12.142921 16.260598 9.936001  C 10.571336 16.199910 11.947526  H 10.346081 16.902023 11.135294  H 10.264245 16.674624 12.885545  C 9.752834 14.943418 11.796499  C 8.638605 14.925370 10.964688  H 8.405349 15.791135 10.352754  C 7.841505 13.783952 10.943675  H 6.967725 13.731080 10.300473  C 8.183705 12.716827 11.761016  H 7.581302 11.814140 11.782242  C 9.322000 12.797895 12.573773  C 15.549587 13.376046 14.223445  H 16.450977 13.168148 14.804260  H 15.638706 12.847349 13.266678  H 14.682013 12.966608 14.746021  C 14.090864 5.906017 20.084568  H 15.132785 5.926061 19.752473  H 13.698656 6.922686 20.097609  H 13.528783 5.338134 19.333112  C 15.449329 8.974787 21.757917  H 15.930678 9.947061 21.608992  H 14.526397 8.949170 21.178659  H 16.133990 8.215848 21.362829  C 13.118546 11.002171 12.120425  H 12.368016 10.207976 12.047383  H 13.051739 11.456436 13.108315  H 14.098043 10.520367 12.022273  C 9.654761 11.673426 13.505767  H 9.161265 11.838180 14.472048  H 10.725736 11.609106 13.696536  H 9.292330 10.720600 13.111584 | Co 8.158614 18.087883 22.862322  Co 12.218594 7.654591 22.387038  Co 12.359254 14.117501 13.323561  O 8.796433 17.646193 20.965549  O 8.722053 16.093065 23.122407  O 11.339991 9.494886 22.832235  O 12.384508 8.576046 20.567386  O 12.414053 12.667457 14.769652  O 11.580216 15.190025 14.930481  N 13.189300 5.671995 22.294221  N 14.276417 15.239627 13.579465  N 7.689163 18.238192 24.966721  N 6.019534 17.437438 22.938590  N 7.696660 20.246378 22.966142  N 10.212419 18.801875 23.781894  N 11.680362 6.914483 24.348920  N 10.142816 6.707075 22.065921  N 14.023530 8.290404 23.606789  N 12.888727 13.244291 11.365676  N 10.169197 13.777628 12.488486  C 11.331358 10.283485 21.812354  C 10.292960 13.779348 20.771288  C 11.423110 19.374607 21.724733  H 11.574481 20.393559 21.352690  H 10.544543 18.939336 21.248427  H 12.302705 18.792672 21.425187  C 7.879566 21.191049 22.019353  C 7.851154 22.552081 22.343333  H 8.005780 23.285969 21.558585  C 7.631578 22.943705 23.655982  H 7.621033 23.996097 23.925212  C 7.421957 21.963793 24.621742  H 7.234999 22.223071 25.659242  C 7.448233 20.627910 24.235499  C 8.074512 20.769924 20.594395  H 8.830053 21.394386 20.108709  H 7.139511 20.911708 20.037933  H 8.365697 19.722839 20.508821  C 7.085512 19.556713 25.235606  H 7.325925 19.895109 26.251000  H 5.995748 19.444862 25.194526  C 8.944929 18.030417 25.709399  H 9.184012 16.963505 25.646231  H 8.820528 18.283027 26.770849  C 10.109387 18.789059 25.126663  C 11.291873 19.374082 23.217828  C 12.295937 19.960950 24.001280  H 13.153544 20.412190 23.511480  C 12.178873 19.963626 25.382130  H 12.942827 20.423877 26.002535  C 11.060655 19.366040 25.960030  H 10.925713 19.340683 27.036735  C 6.735179 17.147287 25.239596  H 6.286843 17.242024 26.237493  H 7.294416 16.206827 25.210387  C 5.659300 17.083568 24.186014  C 4.379032 16.634113 24.493942  H 4.127612 16.368951 25.516045  C 3.446400 16.530037 23.466105  H 2.439955 16.174404 23.668128  C 3.818471 16.900558 22.180305  H 3.114104 16.842779 21.356455  C 5.115684 17.366326 21.941574  C 9.269419 16.440584 20.860149  C 9.232267 15.593920 22.049261  C 9.743724 14.289398 21.952831  H 9.692650 13.686527 22.852460  C 10.329672 14.621266 19.597703  C 9.812676 15.922507 19.681370  H 9.817012 16.582365 18.821230  C 5.524532 17.836752 20.580155  H 4.902007 17.379295 19.807181  H 6.575321 17.611679 20.383113  H 5.396369 18.923732 20.506951  C 10.825842 12.423790 20.708461  C 11.377052 11.922042 19.471841  C 11.394669 12.764025 18.288305  C 10.895181 14.115243 18.356306  C 10.820555 11.591301 21.833580  H 10.419481 11.933694 22.780939  C 11.900841 9.784380 20.565408  C 11.899201 10.617543 19.444965  H 12.333572 10.209551 18.539328  C 11.902877 12.292540 17.064892  H 12.270881 11.277164 16.968738  C 11.960792 13.085897 15.916620  C 11.487900 14.463352 15.992916  C 10.963033 14.919538 17.210967  H 10.611930 15.945124 17.225971  C 10.223355 7.119774 24.461817  H 10.050237 8.197564 24.542125  H 9.819496 6.643810 25.365417  C 9.493525 6.617836 23.241603  C 9.524618 6.297557 20.940192  C 8.207162 5.826452 20.972090  H 7.729943 5.518212 20.047075  C 7.533145 5.746834 22.183462  H 6.512496 5.376919 22.224415  C 8.192729 6.133857 23.345862  H 7.712831 6.070291 24.317372  N 12.076927 15.859275 12.068702  C 12.767423 16.960318 12.764739  H 12.162960 17.224806 13.637443  H 12.851604 17.851995 12.129217  C 14.129269 16.536494 13.254047  C 15.462092 14.801938 14.047775  C 16.528027 15.686635 14.240679  H 17.467819 15.310426 14.632259  C 16.374502 17.028773 13.916378  H 17.195534 17.726810 14.053628  C 15.157777 17.462908 13.399293  H 14.999942 18.498804 13.115442  C 13.095378 5.030008 23.476292  C 13.877080 3.922230 23.787228  H 13.778527 3.445689 24.757524  C 14.773084 3.446400 22.834522  H 15.401122 2.586277 23.048996  C 14.847348 4.088448 21.606939  H 15.526047 3.741222 20.834114  C 14.041593 5.205128 21.357404  C 12.400232 7.740439 25.332681  H 12.391716 7.273436 26.326037  H 11.865004 8.692587 25.413487  C 13.813169 8.060977 24.918306  C 14.813048 8.204690 25.873391  H 14.599786 7.998941 26.917623  C 16.074777 8.625850 25.459627  H 16.878439 8.752076 26.179668  C 16.285593 8.880548 24.113156  H 17.252941 9.215357 23.751319  C 15.240297 8.696848 23.198402  C 12.027583 5.483253 24.443047  H 11.127487 4.900829 24.214578  H 12.315361 5.219479 25.467842  C 10.295671 6.315048 19.656613  H 9.622745 6.408987 18.800120  H 10.844423 5.372121 19.540468  H 11.021592 7.130771 19.646545  C 12.749917 14.142405 10.369707  C 12.771743 13.776944 9.028001  H 12.640761 14.531443 8.258698  C 12.969975 12.438309 8.702424  H 12.988731 12.119767 7.664024  C 13.146135 11.519575 9.726872  H 13.314828 10.468675 9.513370  C 13.102117 11.947626 11.058595  C 12.674113 15.602496 10.745919  H 13.704838 15.975935 10.761262  H 12.150368 16.164991 9.963079  C 10.627424 16.114548 11.995938  H 10.396115 16.860095 11.223994  H 10.324374 16.530735 12.962391  C 9.814784 14.864838 11.773258  C 8.707290 14.884389 10.933225  H 8.470677 15.780117 10.367300  C 7.915662 13.740497 10.847245  H 7.042161 13.719735 10.201514  C 8.264392 12.630876 11.600801  H 7.670281 11.722629 11.567787  C 9.404860 12.673386 12.416273  C 15.617336 13.336534 14.313737  H 16.431815 13.151028 15.018331  H 15.861820 12.812913 13.381154  H 14.691755 12.906734 14.704956  C 14.092106 5.877632 20.018843  H 15.122790 5.939749 19.657374  H 13.655503 6.876390 20.041460  H 13.534616 5.282346 19.285147  C 15.472377 8.948469 21.739266  H 15.911740 9.940731 21.590992  H 14.543134 8.887263 21.171224  H 16.184529 8.220347 21.334389  C 13.327925 10.963732 12.166321  H 12.782263 10.035031 11.974357  H 13.029412 11.360516 13.136996  H 14.391777 10.700543 12.216432  C 9.781560 11.473159 13.231704  H 8.985959 11.241060 13.949285  H 10.706220 11.644851 13.783793  H 9.897399 10.592558 12.590566 |

## S3. Infrared spectroscopy

| **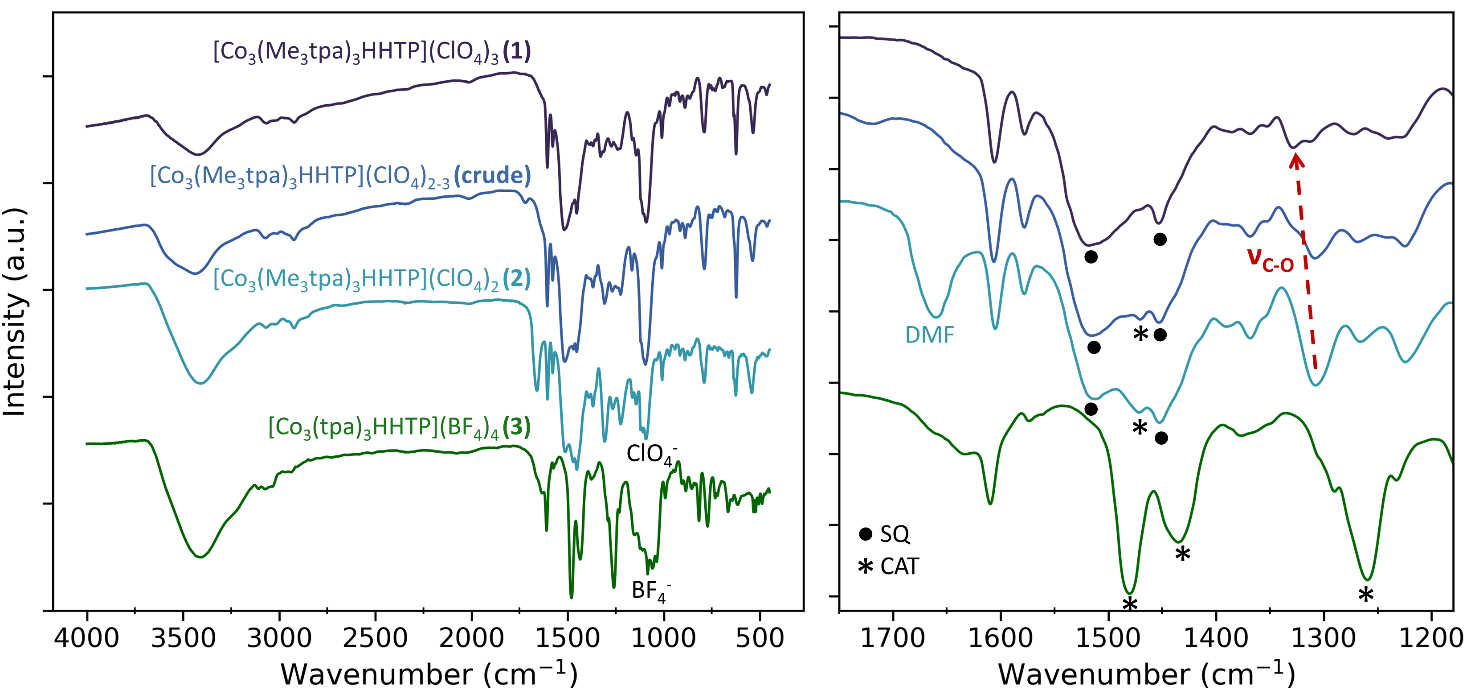** |
| --- |
| **Figure S8.** FT-IR spectra of crude [Co_3_(Me_3_TPA)_3_HHTP](ClO_4_)_2-3_, **1,** **2** and **3** obtained as crystals (**1**, **2**) or powders (crude, **3**). |

In the study of metal complexes involving dioxolene ligands, infrared spectroscopy has been widely employed to characterize the different oxidation states, most particularly using the C-O and C=C catecholate/semiquinone vibrational bands.^[13,14]^ Therefore, to investigate the oxidation state of HHTP within the [Co_3_(Me_3_TPA)_3_HHTP](ClO_4_)_2-3_ complexes, solid state infrared spectra in KBr (1% in weight) were recorded and are presented in **Figure S3**. To have a better insight of the attribution of the vibrational bands, comparison with the reported TPA complex is also presented (**Figure S3**). An IR analysis is proposed here, based on comparisons with previously reported theoretical^[13,14]^ and experimental^[15–19]^ studies on analogue complexes.

The infrared spectrum of [Co_3_(TPA)_3_HHTP](BF_4_)_4_ (**3**) exhibits distinct bands characteristic of the catecholate form (indicated by a * on the spectrum), including an intense catecholate aromatic skeletal vibration at 1260 cm^-1^, and two C-O vibrational bands at 1447 and 1482 cm^-1^. Additionally, less intense bands at 1573 and 1610 cm^-1^ are attributed to C=C/C=N stretches of the dioxolene moieties and TPA ligands.^[13,14]^ The absence of semiquinone bands suggests that the remaining single electron in the (cat-cat-sq)^5•-^ form of HHTP is not localized on the OCCO moiety, but is rather delocalized across the central ligand on the IR timescale, as suggested by the reported SXRD structure.^[3]^ On the other hand, the infrared spectra of [Co_3_(Me_3_TPA)_3_HHTP](ClO_4_)_3_ (**1)** and [Co_3_(Me_3_TPA)_3_HHTP](ClO_4_)_2_ (**2)** reveal significant shifts or disappearance of these bands, alongside an increase in the semiquinone characteristic stretching frequencies (indicated by a ● on the spectrum). The two C=C/C=N stretching frequencies are more intense, and their energy difference is reduced compared to the TPA complex (**Table S4**), as usually observed in the Me_n_TPA complex family.^[14–17]^ For **2**, the spectrum displays three bands in the 1400-1550 cm^-1^ region (1514, 1472 and 1453 cm^-1^), while for **1,** the band at 1453 cm^-1^ is more intense, accompanied by the disappearance of the 1472 cm^-1^ vibrational band. Compared to **3,** which features a strong catecholate C-O band at 1482 cm^-1^, it suggests that

| Table S1. Infra-red data and tentative of attribution for the studied complexes. | | | | | |
| --- | --- | --- | --- | --- | --- |
| Assignment ν /cm^-1^ | **1** | **Crude (1+2)** | **2** | **3** | **Ref.** |
| TPA - ν(C=C) | 1606 | 1606 | 1605 | 1610 | ^[14]^ |
| TPA - ν(C=N) | 1578 | 1578 | 1578 | 1574 | ^[14]^ |
| SQ - ν(C=O) | 1515  1453 | 1515  1453 | 1514  1453 | - | ^[14]^ |
| CAT - ν(C-O) + δ(C-H) diox | - | 1471 | 1472 | 1480  1434 | ^[14]^ |
| CAT - Skeletal diox | - | 1268 | 1266 | 1260 | ^[13,14]^ |
| ν(C-O)/ ν(C=O)^a^ | 1328  1312  1272  1240  1227 | 1308  1268  1243  1225 | 1308  1266  1224 | 1290  1232 | ^[13,14,18]^ |
| ^a^ Attempted assignments, with probably ν_C=C_, ν_C-C_ and δ_C-H_ contributions based on literature. ^[14]^ | | | | | |

the latter band is associated with the catecholate form, in agreement with the (cat-sq-sq)^4-^ and (sq-sq-sq)^3•-^ redox-state of HHTP for **2** and **1**, respectively. Therefore, the two bands at 1514 and 1453 cm^-1^ observed in **1** and **2**, and absent in **3**, can be attributed to C=O stretching frequencies from the semiquinone groups.^[14]^ In the 1200-1300 cm^-1^ range, three bands are observed for **2** at 1308, 1266 and 1224 cm^-1^, while they decrease in intensity with slight shifts for **1,** with the appearance of additional bands at 1328 and 1240 cm^-1^. In the literature, the presence of three distinct vibrational bands in this region is characteristic of catecholate groups, such as the intense catecholate aromatic skeletal vibration at 1260 cm^-1^ for **3**. While for semiquinone groups, the identification of distinctive bands is more complicated.^[13]^ In addition, it is worth noting that the attribution of the bands is less trivial in these trinuclear systems than for the monomers. Consequently, in absence of computational data, we attempted to attribute these bands to C-O/C=O stretching modes, with probably ν_C=C_, ν_C-C_ and δ_C-H_ contributions.^[14]^ For **1** and **2**, the IR spectra are similar to their reported Ni analogues, for which the authors highlighted a blue-shift of the C-O band going from the (cat-sq-sq)^4-^ to the (sq-sq-sq)^3•-^ redox-state of HHTP due to an increase on the semiquinone character of the ligand. ^[18]^ This blue-shift can also be suggested here from **2** and **1** (red arrows, **Figure S3**). Finally, the spectrum of the crude powder from the initial synthesis in MeOH displays features of both **1** and **2**, confirming the obtention of a mixture of redox-state during the initial synthesis.

## S4. Single-Crystal X-ray Analysis

| 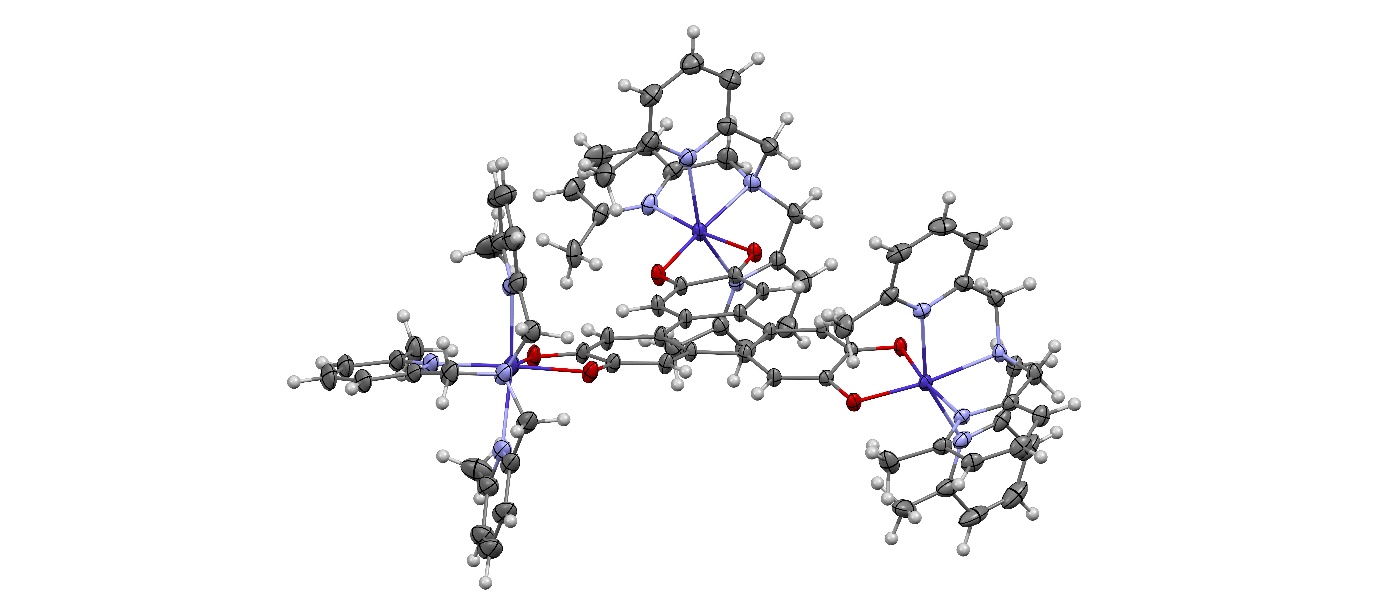 |
| --- |
| **Figure S9.** An ORTEP drawing of compound **2.** Thermal ellipsoids are shown at the 30% level (hydrogen atoms and solvent are omitted for clarity). |

X-ray diffraction data for compounds **1** and **2** were collected by using a VENTURE PHOTONIII c7 Bruker diffractometer with Micro-focus IuS source Mo Kα radiation. Crystals were selected under optical microscope and glued in paratone oil. A crystal was mounted on a CryoLoop (Hampton Research) with Paratone-N (Hampton Research) as cryoprotectant and then placed in a nitrogen-gas stream at 200 K. The temperature of the crystal was maintained at the selected value by means of a cooling device by a 700 series Cryostream cooling device to with an accuracy of ±1K. Data reduction was accomplished using SAINT V7.53a. The substantial redundancy in data allowed a semi-empirical absorption correction (SADABS V2.10) to be applied, on the basis of multiple measurements of equivalent reﬂections. The structures were solved by direct methods using SHELXS-97^[20]^ and refined against F2 by full-matrix least-squares techniques using SHELXL-2018^[21]^ with anisotropic displacement parameters for all non-hydrogen atoms^[22]^. All calculations were performed by using the Crystal Structure crystallographic software package WINGX. ^[23]^

CCDC 2350894 & 2350895 contain the supplementary crystallographic data for this paper. These data can be obtained free of charge from the Cambridge Crystallographic Data Centre and Fachinformationszentrum Karlsruhe via http://www.ccdc.cam.ac.uk/structures/.

The crystal data collection and refinement parameters are given in **Table S2**, and a selection of bond distances and angles in **Table S4**. The asymmetric unit of **1** contains 5 molecules of toluene and 4 molecules of H_2_O while compound **2** contains 3 molecules of dimethylformamide and 2 of H_2_O.

| **Table S2.** Crystallographic data and structure refinement details. | | |
| --- | --- | --- |
| **Compound** | **1** | **2** |
| T, K | 200(1) | 200(1) |
| CCDC | 2350895 | 2350894 |
| Empirical Formula | C_81_ H_78_ Co_3_ N_12_ O_6_, 3(Cl O_4_), 3.27(C_7_ H_8_), 3.483(O) | C_81_ H_78_ Co_3_ N_12_ O_6_, 2(Cl O_4_), 2,5(C_3_ H_7_ N O), 2(H_2_ O) |
| *M_r_* | 2147.71 | 1907.99 |
| Crystal size, mm^3^ | 0.14 x 0.05 x 0.04 | 0.14 x 0.05 x 0.04 |
| Crystal system | monoclinic | triclinic |
| Space group | *P* 2_1_*/n* | *P -*1 |
| a, Å | 10.7662(6) | 8.8137(10) |
| b, Å | 33.353(2) | 20.277(3) |
| c, Å | 31.0764(19) | 27.600(4) |
| α, ° | 90 | 101.288(5) |
| β, ° | 96.999(3) | 90.871(5) |
| γ, ° | 90 | 102.294(5) |
| Cell volume, Å^3^ | 11076.0(12) | 4717.9(10) |
| Z ; Z’ | 4 ; 1 | 2 ; 1 |
| Radiation type ; wavelength Å | Mo Kα ; 0.71073 | Mo Kα ; 0.71073 |
| F_000_ | 4462 | 1986 |
| µ, mm^–1^ | 0.587 | 0.646 |
| range, ° | 1.799 - 24.451 | 2.005 - 31.026 |
| Reflection collected | 396 231 | 366 309 |
| Reflections unique | 18 302 | 30 089 |
| R_int_ | 0.1359 | 0.0829 |
| GOF | 1.422 | 1.045 |
| Refl. obs. (*I*>2(*I*)) | 10 457 | 17 806 |
| Parameters ; restraints | 1032 ; 11 | 1124 ; 25 |
| wR_2_ (all data) | 0.3988 | 0.2514 |
| R value (I > 2(I)) | 0.1230 | 0.0740 |
| Largest diff. peak and hole (e-.Å^-3^) | 2.085 ; -1.069 | 1.998 ; -0.924 |

| \| **Table S3.** Geometric parameters obtained with Shape on structures of **1** and **2.** \| \| \| \| \| \| \| \| --- \| --- \| --- \| --- \| --- \| --- \| --- \| \| Structure [ML6] \| \| **HP-6** \| **PPY-6** \| **OC-6** \| **TPR-6** \| **JPPY-6** \| \| **1** \| Co1 \| 30.771 \| 26.406 \| 1.366 \| 14.779 \| 29.873 \| \| Co2 \| 28.409 \| 26.148 \| 1.562 \| 14.699 \| 28.271 \| \| Co3 \| 29.429 \| 27.219 \| 1.205 \| 15.216 \| 29.676 \| \| **2** \| Co1 \| 29.406 \| 25.381 \| 1.409 \| 14.153 \| 28.507 \| \| Co2 \| 30.037 \| 25.154 \| 1.586 \| 13.815 \| 28.569 \| \| Co3 \| 30.820 \| 26.057 \| 1.669 \| 13.880 \| 28.240 \| \| *HP-6 Hexagon, PPY-6 Pentagonal pyramid, OC-6 Octahedron, TPR-6 Trigonal prism, JPPY-6 Johnson pentagonal pyramid J2* \| \| \| \| \| \| \|   **Table S4**. Selected bond distances (Å) and angles (°) in **1** and **2**. | | | | | |
| --- | --- | --- | --- | --- | --- | --- | --- | --- | --- | --- | --- | --- | --- | --- | --- | --- | --- | --- | --- | --- | --- | --- | --- | --- | --- | --- | --- | --- | --- | --- | --- | --- | --- | --- | --- | --- | --- | --- | --- | --- | --- | --- | --- | --- | --- | --- | --- | --- | --- | --- | --- | --- | --- | --- | --- | --- | --- | --- | --- | --- | --- | --- | --- | --- |
| **1** | | | | | |
| **Co1** | | **Co2** | | **Co3** | |
| Co1-O2  Co1-O1  Co1-N1  Co1-N3  Co1-N2  Co1-N4  **C1 -O2**  **C18-O1**  **C1-C18**  $\hat{O1Co1O2}$ | 1.975(5)  2.051(5)  2.065(7)  2.115(7)  2.181(7)  2.230(9)  **1.309(8)**  **1.274(8)**  **1.458(10)**  81.4(2) | Co2-O4  Co2-O3  Co2-N5  Co2-N6  Co2-N7  Co2-N8  **C6-O3**  **C7-O4**  **C6-C7**  $\hat{O3Co2O4}$ | 2.000(5)  2.144(5)  2.120(6)  2.145(8)  2.163(6)  2.309(9)  **1.259(9)**  **1.285(8)**  **1.454(11)**  79.2(2) | Co3-O5  Co3-O6  Co3-N9  Co3-N10  Co3-N11  Co3-N12  **C12-O5**  **C13-O6**  **C12-C13**  $\hat{O5Co2O6}$ | 1.993(6)  2.118(5)  2.100(7)  2.136(9)  2.151(7)  2.267(7)  **1.322(10)**  **1.272(8)**  **1.463(10)**  79.9(2) |
| **2** | | | | | |
| **Co1** | | **Co2** | | **Co3** | |
| Co1-O1  Co1-O2  Co1-N1  Co1-N2  Co1-N4  Co1-N3  **O1-C1**  **O2-C2**  **C1-C2**  $\hat{O1Co1O2}$ | 1.978(2)  2.113(2)  2.137(3)  2.205(2)  2.217(3)  2.274(3)  **1.311(3)**  **1.313(3)**  **1.440(4)**  81.89(8) | Co2-O4  Co2-O3  Co2-N6  Co2-N5  Co2-N8  Co2-N7  **O3-C7**  **O4-C8**  **C7-C8**  $\hat{O3Co2O4}$ | 1.993(2)  2.109(2)  2.131(3)  2.144(3)  2.248(3)  2.276(3)  **1.257(3)**  **1.303(3)**  **1.473(4)**  80.05(8) | Co3-O5  Co3-O6  Co3-N9  Co3-N10  Co3-N11  Co3-N12  **O5-C13**  **O6-C14**  **C13-C14**  $\hat{O5Co2O6}$ | 2.012(3)  2.119(2)  2.127(4)  2.153(3)  2.258(4)  2.278(3)  **1.317(4)**  **1.269(4)**  **1.466(5)**  80.13(10) |

Calculation using the Shape software^[24]^ were done on the structures to determine the geometry around the cobalt centers. Results are shown in **Table S3**, which confirmed the octahedral environment of the three cobalt centers in the two complexes.

## S5. Theoretical calculations


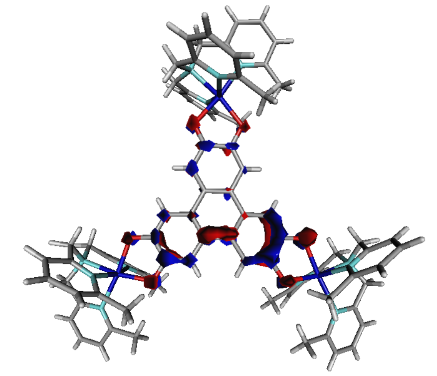

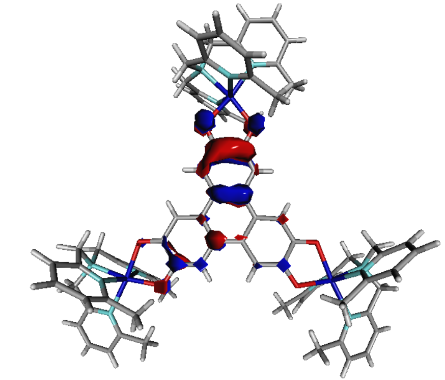

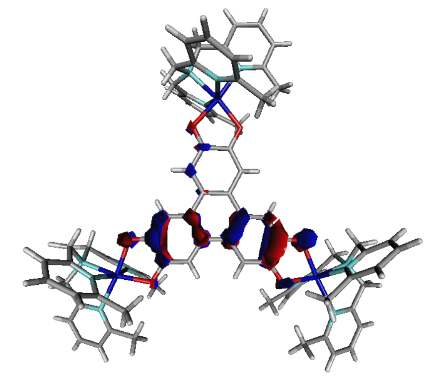


Occ. 1.65 1.02 0.32

E(eV/cm^-1^) 0.00 / 0.00 1.96 / 15817 2.87 / 23137

**Figure S10**. First line: representation of the natural state average active MO obtained from CAS(3/3) calculations of the ground and first excited doublet states and the lowest quartet state of complex **1**. MO issued from the bonding (left) and anti-bonding (right) interaction between MO localized on the OCCO moieties close to Co2 and Co3. Center: magnetic MO mainly localized on the OCCO moiety close to Co1. Second line: occupation number of the natural MO. Third line: relative energy (eV/cm^-1^) of the canonical MOs compared to the lowest one. Orientation and numbering are the same as for Scheme 2 (left).

**Table S5**. Transition energies from the doublet ground state to the first excited doublet and lowest quartet states evaluated at the CAS(3/3)SCF and CAS(3/3)+NEVPT2 levels of calculation on state average MO for complex **1**.

| E (eV/cm^-1^) | First excited doublet state | Lowest quartet state |
| --- | --- | --- |
| CAS(3/3)SCF | 0.541 / 4362 | 0.577 / 4653 |
| CAS(3/3)+NEVPT2 | 0.501 / 4039 | 0.789 / 6365 |


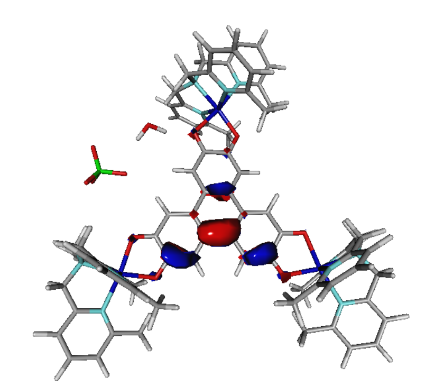

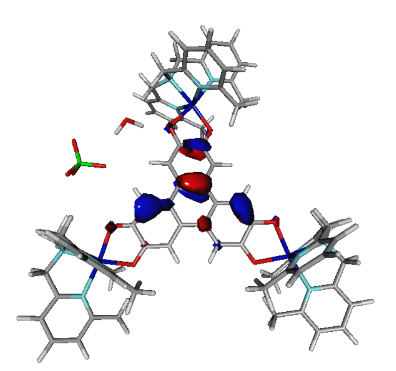

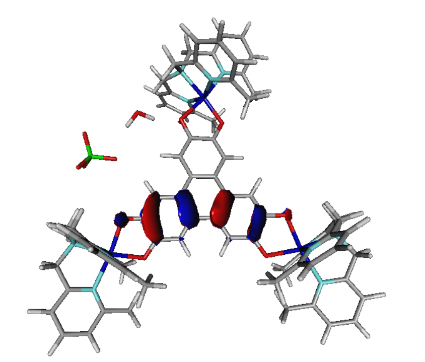


Occ. 1.88 1.95 0.16

E(eV/cm^-1^) 1.99 / 16029 0.00 / 0.00 5.24 / 42285

**Figure S11**. First line: representation of the natural state average active MO obtained from CAS(4/3) calculations of the singlet ground state and lowest triplet state of complex **2**. MO issued from the bonding (left) and anti-bonding (right) interaction between MO localized on the OCCO moieties close to Co2 and Co3. Center: MO mainly localized on the OCCO moiety close to Co1. Second line: occupation number of the natural MOs. Third line: relative energy (eV/cm^-1^) of the canonical MOs compared to the lowest one. Orientation of the molecule and numbering of the atoms are the same as in Scheme 2 (right).

**Table S6**. Transition energies from the singlet ground state to the lowest triplet state evaluated at the CAS(4/3)SCF and CAS(4/3)+NEVPT2 levels of calculation on state average MO for complex **2**.

| E (eV/cm^-1^) | Triplet state |
| --- | --- |
| CAS(4/3)SCF | 0.712 / 5746 |
| CAS(4/3)+NEVPT2 | 0.284 / 2290 |

It is worth noting that the TD-DFT calculations on the *optimized structures* of the complexes for the sq-sq-sq (complex 1) and the cat-sq-sq (complex 2) states of HHTP show that the ground electronic states are those with the high spin configurations i.e. the quadruplet (S = 3/2) and the triplet (S = 1) for sq-sq-sq and cat-sq-sq, respectively. While the *ab initio* calculations performed on the *crystallographic structures* of the two complexes show that the ground electronic states are the low spin ones i.e. the doublet (S =1/2) and singlet (S = 0) for sq-sq-sq and cat-sq-sq, respectively. The origin of this difference is the *geometrical* structure of the bridging HHTP that governs its electronic structure. The optimized structures for complexes **1** and **2** lead to perfectly flat HHTP (see the coordinates in section S2), while for the crystallographic structures, HHTP is distorted. Since the TD-DFT calculations reproduce very well the experimental UV-visible electronic spectra, one concludes that the structure of the complexes in solution is different from the solid state. The comparison of the electronic behavior between the two situations (solution and solid state) needs the analysis of the magnetic data (that are studied on the solid state). This is out of the scope of this paper and has, at any rate, already been investigated theoretically by some of us in a previous paper.^[25]^

## S6. Electrochemistry

| **Table S7.** Cyclic voltammetry data of **1**, **2** and **3**. All potentials are versus ferrocene couple. | | | | | | | | | |
| --- | --- | --- | --- | --- | --- | --- | --- | --- | --- |
| **Complex** | **OCP** | **E_1/2_ /V (ΔE_p_ /mV)^a^** | | | | | | | **Ref.** |
|  |  | **I** | **II** | **III** | **IV** | **V** | **VI** | **VII** |  |
| **1** (1mM CH_2_Cl_2_) | -0.32 | -1.11  (100) | -0.84 (80) | -0.43 (60) | 0.18  (80) | 0.37  (60) | 0.52  (80) | 0.87  (46) | This work |
|  |  | **I’** | **II’** | **III’** | - | - | - | - |  |
| **2** (1mM DMF) | -0.35 | -0.75 (100) | -0.34 (80) | 0.12^b^ | - | - | - | - |  |
|  |  | **I’’** | **II”** | **III’’** | - | - | - | - |  |
| **3** (1mM CH_3_CN) | -0.34 | -0.37 (100) | 0.02 (80) | 0.39 (100) | - | - | - | - |  |
|  | -0.05 | -0.33 | 0.05 | 0.41 | - | - | - | - | ^[3]^ |

Cyclic voltammetry data for each redox process are displayed in **Table S7**. To have a better understanding on the influence of the Me_3_TPA ligand on the electronic properties, the TPA complex in CH_3_CN (**3**) was also studied as a comparison (**Figure S13 and S15**). For **1** in CH_2_Cl_2_, an additional oxidation process (VII) was observed by scanning at higher potentials. Comparisons with similar studied compounds in our group^[26]^ suggest partial decoordination of the compound at high potentials. In DMF, additional non-reversible redox-processes were also observed by increasing the potential window, that was attributed to partial decomposition of the compound, or additional oxidation of the complex that were not reversible (**Figure S13b**). Finally, measurements at different scan rates (**Figure S12 and S14**) confirmed the good reversibility of most redox-processes (II, III, IV and V in DCM, and II’ in DMF), and Randles–Sevcik plots corroborates with diffusion-controlled processes in solution. In DCM, process I was not reversible upon oxidation, which can be attributed to the adsorption of the neutral [Co^II^(cat-cat-cat)]^0^ on the electrode after reduction. On the other hand, VI was not reversible upon reduction, and the peak observed for some samples also suggest the possible adsorption of the +6 charged form. In DMF, only II’ was fully reversible, while I’ was reversible mostly upon reduction. For III’, only the anodic potential is visible, for this reason the half-potential was determined using the DPV measurement upon oxidation.

^a^E_1/2_ potentials were calculated with the average of the peak potentials measured on CV ^b^ Measured from Differential Pulse Voltammetry (DPV) in oxidation

| **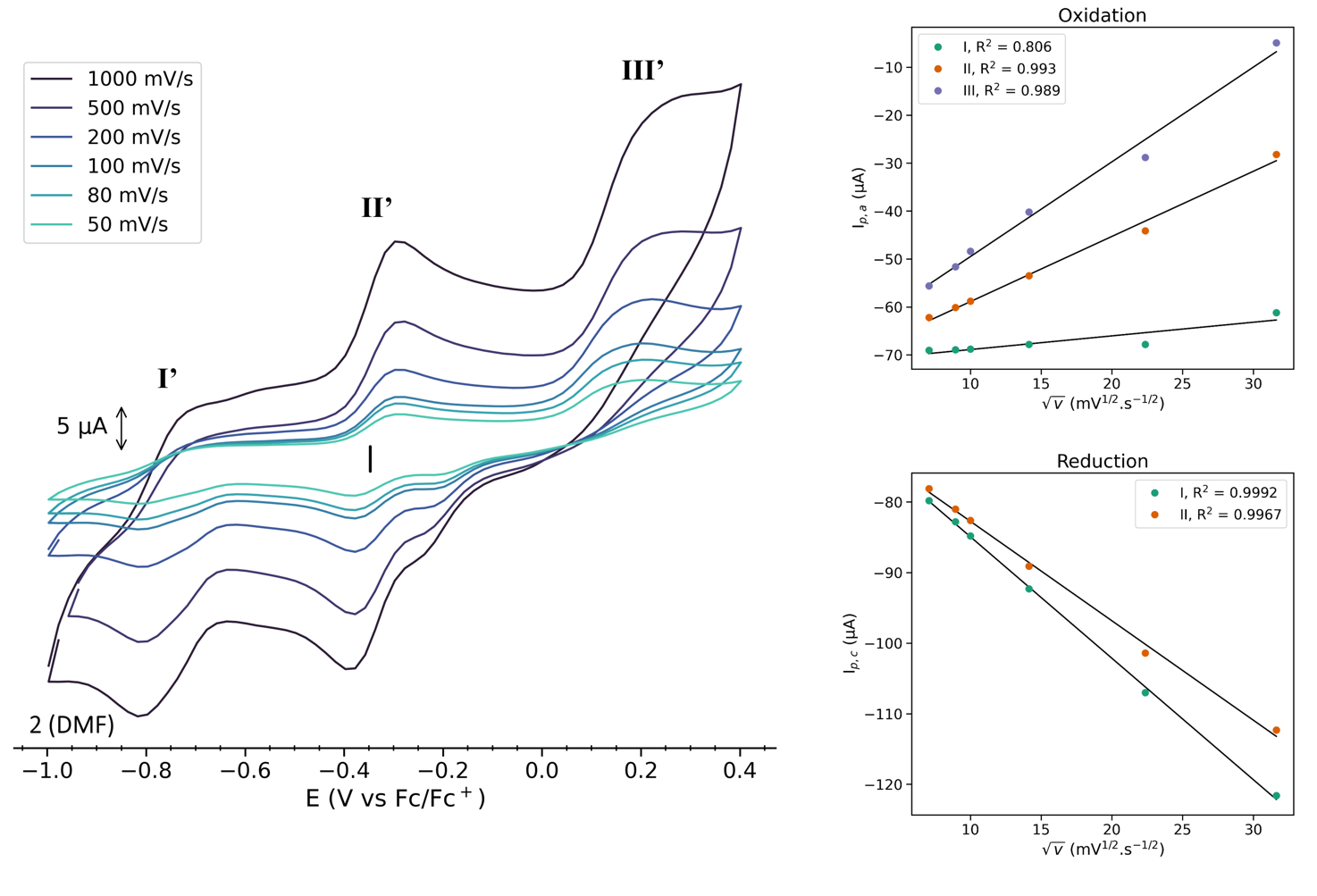** | |
| --- | --- |
| **Figure S12.** Cyclic voltammetry of **2** recorded in 1 mM DMF with 0.1 mM TBAPF_6_ at different scan rates, and associated Randles–Sevcik plots. | |
| 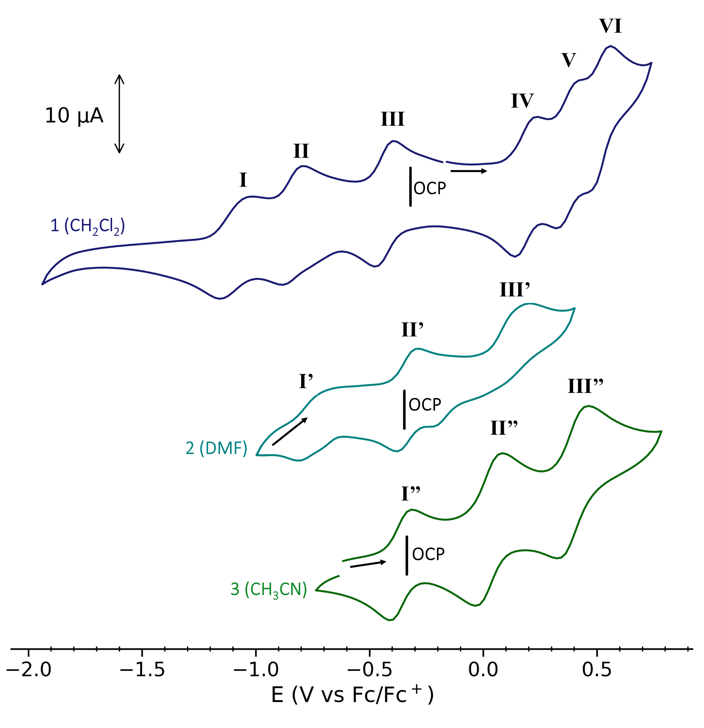  (a) | 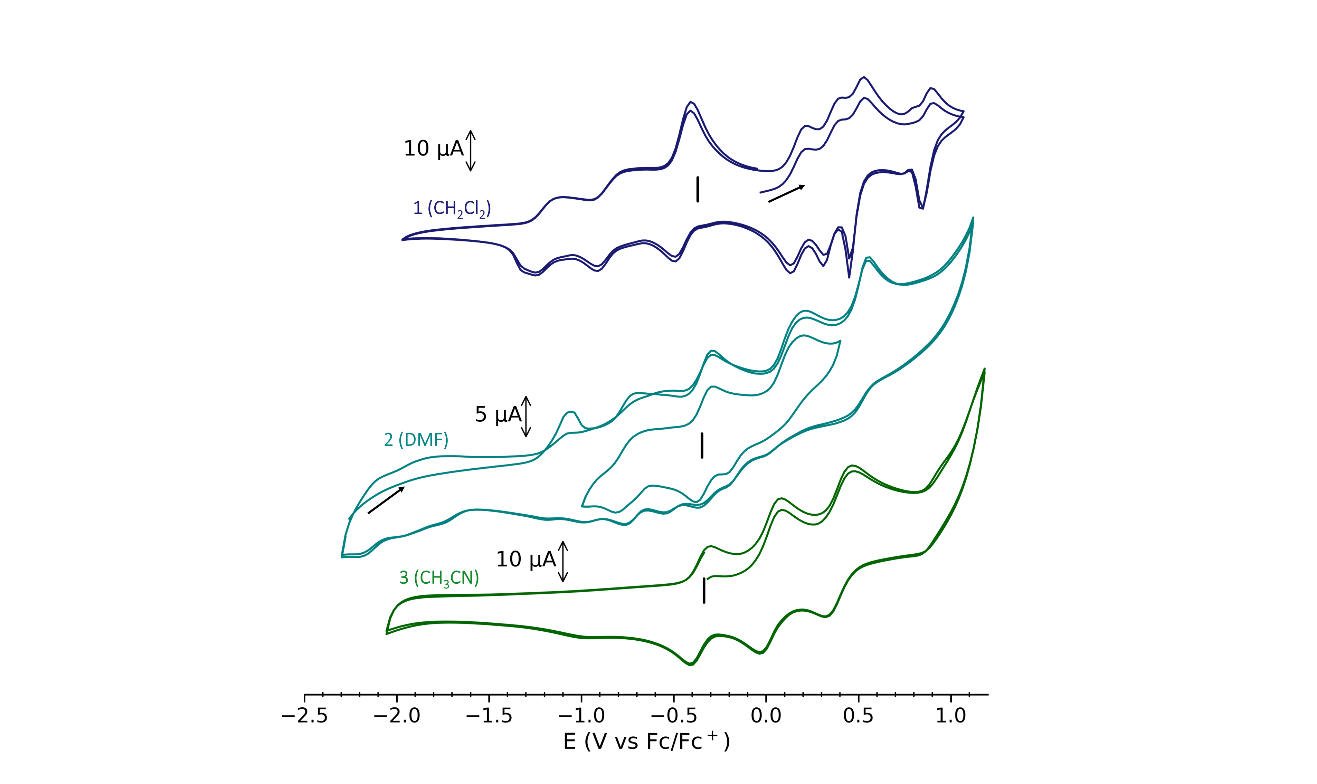 (b) |
| **Figure S13.** (a) Partial and (b) full-scan cyclic voltammograms of **1** (1mM DCM, 100 mV.s^-1^), **2** (1mM DMF, 100 mV.s^-1^) and **3** (1mM CH_3_CN, 100 mV.s^-1^ and 200 mV.s^-1^) with 0.1 mM TBAPF_6_. | |

| **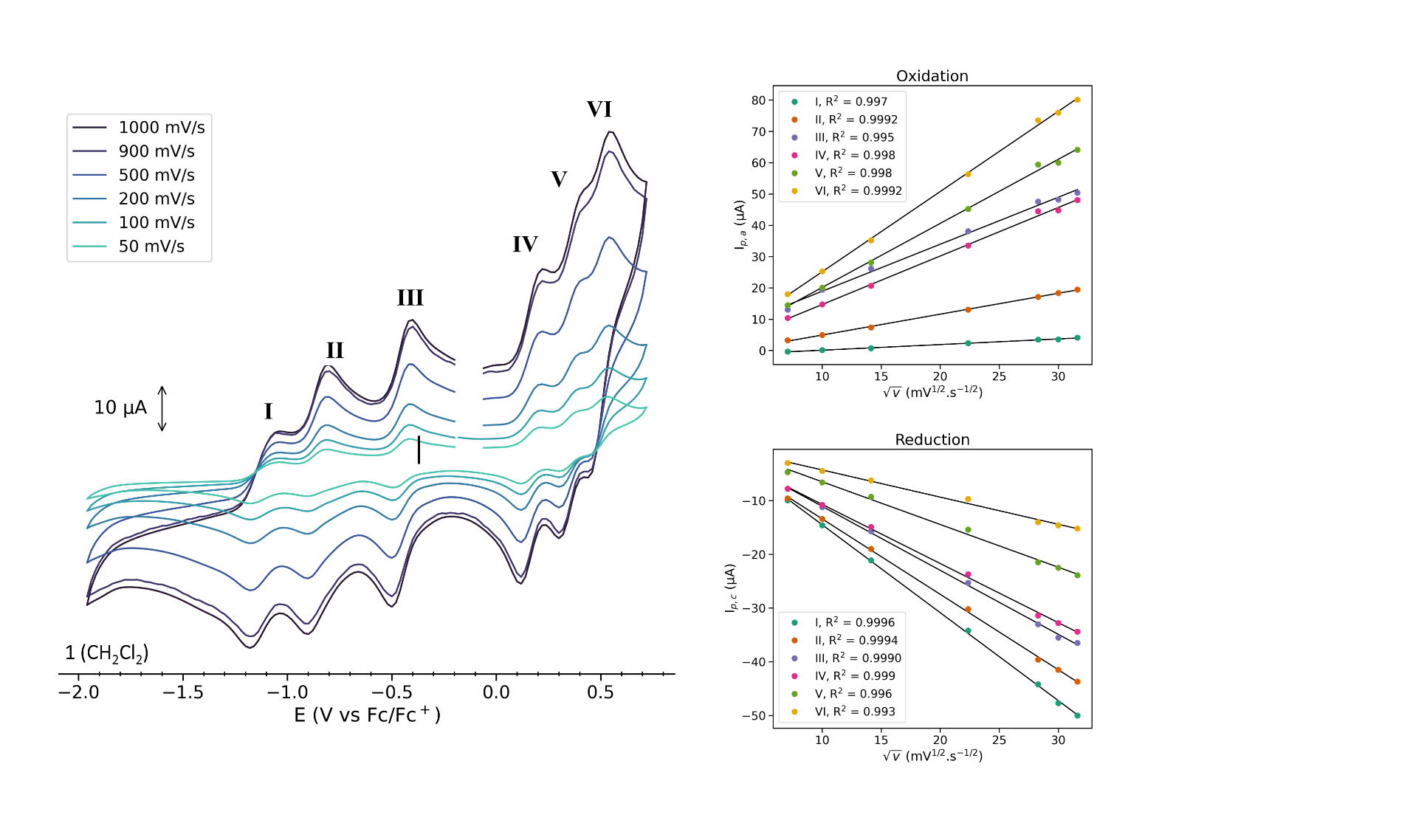** |
| --- |
| **Figure S14.** Cyclic voltammetry of **1** recorded in 1 mM DCM with 0.1 mM TBAPF_6_ at different scan rates, and associated Randles–Sevcik plots. |
| **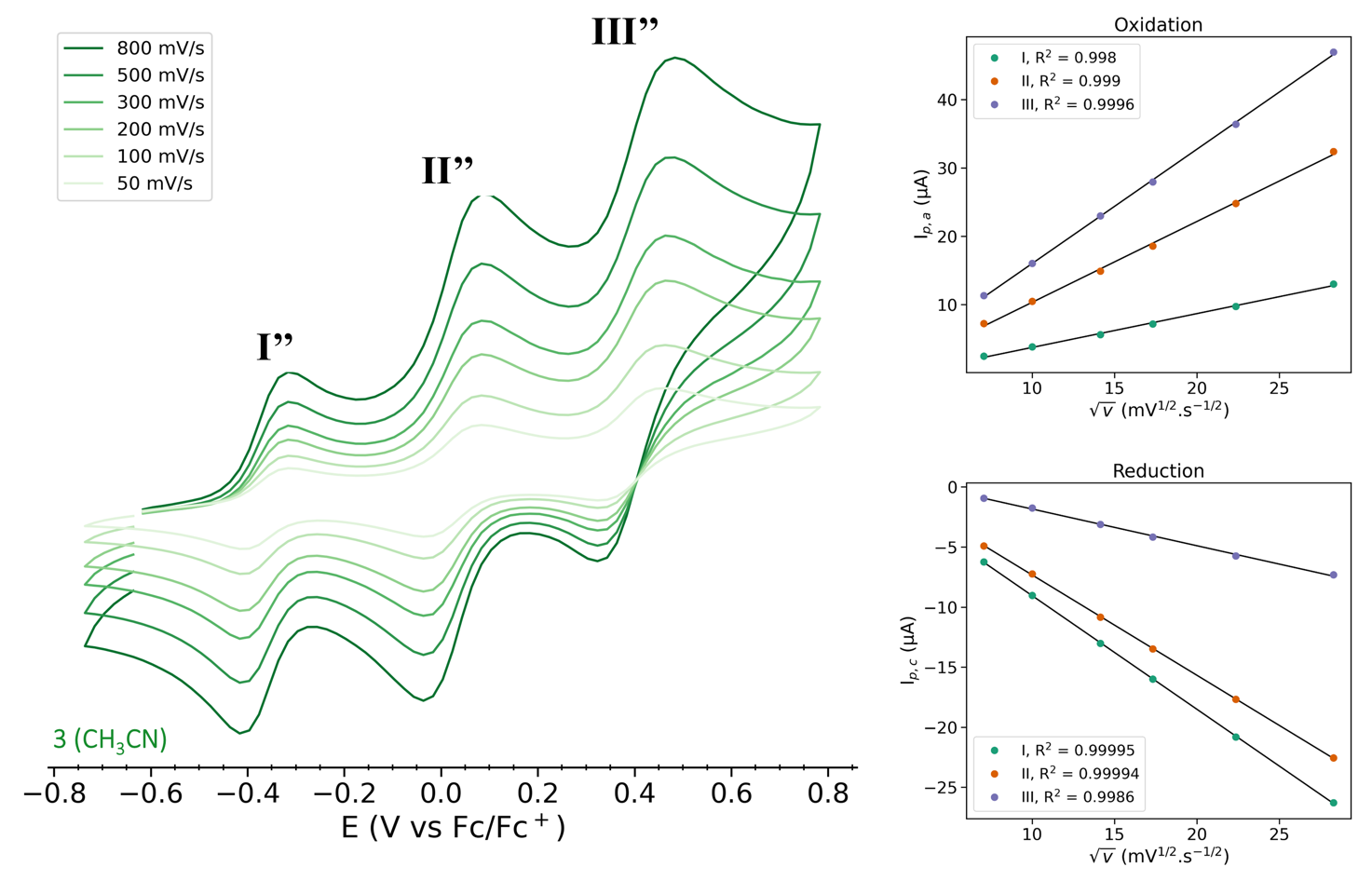** |
| **Figure S15.** Cyclic voltammetry of **3** recorded in 1 mM CH_3_CN with 0.1 mM TBAPF_6_ at different scan rates, and associated Randles–Sevcik plots. S7. Magnetic characterizations **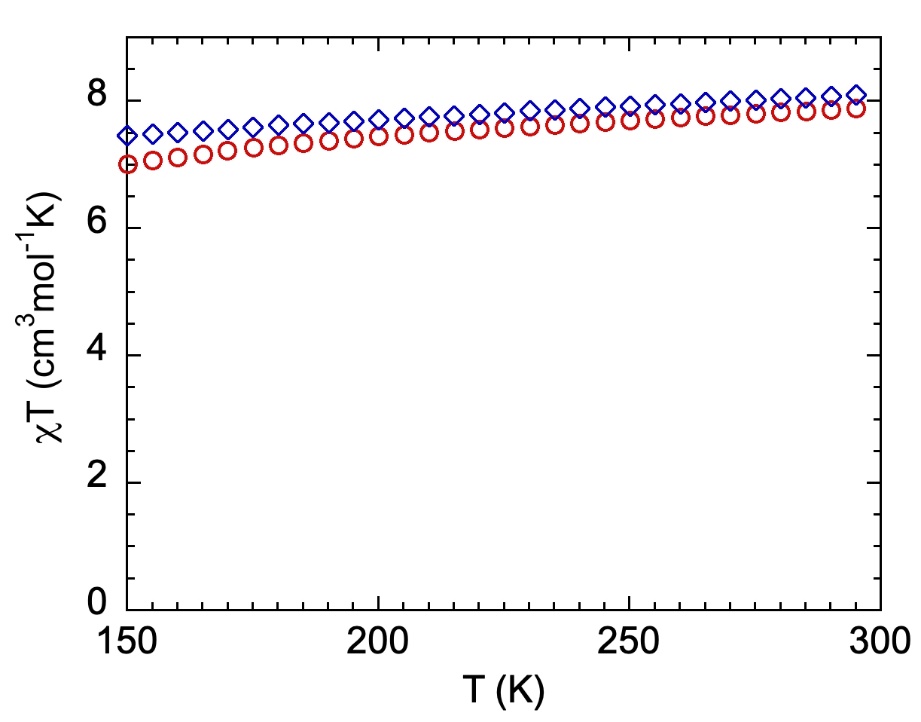**  **Figure S16.** Thermal variation of the χT product for **1** (◇) and **2** (⭘) in the 300-150 K range. The χT values at room temperature are consistent with the presence of three high spin Co(II) ions with S = 3/2 and relatively large *g*-values as expected for octahedral high spin species, where *g* may vary from 2.2 to 2.6. Here, the calculated average *g*-value is close to 2.4. |

## References

[1] R. Janicki, *Journal of Molecular Structure* **2013**, *1036*, 35–41.

[2] A. Beni, A. Dei, S. Laschi, M. Rizzitano, L. Sorace, *Chem. – Eur. J.* **2008**, *14*, 1804–1813.

[3] Y. Suenaga, H. Inada, M. Inomata, R. Yamaguchi, T. Okubo, M. Maekawa, T. Kuroda-Sowa, *Chem. Lett.* **2014**, *43*, 562–564.

[4] M. J. Frisch, G. W. Trucks, H. B. Schlegel, G. E. Scuseria, M. A. Robb, J. R. Cheeseman, G. Scalmani, V. Barone, B. Mennucci, G. A. Petersson, H. Nakatsuji, M. Caricato, X. Li, H. P. Hratchian, A. F. Izmaylov, J. Bloino, G. Zheng, J. L. Sonnenberg, M. Hada, M. Ehara, K. Toyota, R. Fukuda, J. Hasegawa, M. Ishida, T. Nakajima, Y. Honda, O. Kitao, H. Nakai, T. Vreven, J. A. M. Jr, J. E. Peralta, F. Ogliaro, M. Bearpark, J. J. Heyd, E. Brothers, K. N. Kudin, V. N. Staroverov, R. Kobayashi, J. Normand, K. Raghavachari, A. Rendell, J. C. Burant, S. S. Iyengar, J. Tomasi, M. Cossi, N. Rega, M. J. Millam, M. Klene, J. E. Knox, J. B. Cross, V. Bakken, C. Adamo, J. Jaramillo, R. Gomperts, R. E. Stratmann, O. Yazyev, A. J. Austin, R. Cammi, C. Pomelli, J. W. Ochterski, R. L. Martin, K. Morokuma, V. G. Zakrzewski, G. A. Voth, P. Salvador, J. J. Dannenberg, S. Dapprich, A. D. Daniels, Ö. Farkas, J. B. Foresman, J. V. Ortiz, J. Cioslowski, D. J. Fox, *Gaussian 09, Revision D.01*, Gaussian, Inc., Wallingford CT, **2009**.

[5] A. D. Becke, *The Journal of Chemical Physics* **1993**, *98*, 5648–5652.

[6] K. Burke, J. P. Perdew, W. Yang, in *Electronic Density Functional Theory: Recent Progress and New Directions* (Eds.: J.F. Dobson, G. Vignale, M.P. Das), Plenum, New York, **1998**.

[7] D. Andrae, U. Häußermann, M. Dolg, H. Stoll, H. Preuß, *Theoret. Chim. Acta* **1990**, *77*, 123–141.

[8] A. Höllwarth, M. Böhme, S. Dapprich, A. W. Ehlers, A. Gobbi, V. Jonas, K. F. Köhler, R. Stegmann, A. Veldkamp, G. Frenking, *Chemical Physics Letters* **1993**, *208*, 237–240.

[9] P. C. Hariharan, J. A. Pople, *Theoret. Chim. Acta* **1973**, *28*, 213–222.

[10] W. J. Hehre, R. Ditchfield, J. A. Pople, *The Journal of Chemical Physics* **1972**, *56*, 2257–2261.

[11] A. V. Marenich, C. J. Cramer, D. G. Truhlar, *J. Phys. Chem. B* **2009**, *113*, 6378–6396.

[12] M. Strohalm, D. Kavan, P. Novák, M. Volný, V. Havlíček, *Anal. Chem.* **2010**, *82*, 4648–4651.

[13] F. Rupp, K. Chevalier, M. Graf, M. Schmitz, H. Kelm, A. Grün, M. Zimmer, M. Gerhards, C. van Wüllen, H.-J. Krüger, R. Diller, *Chemistry – A European Journal* **2017**, *23*, 2119–2132.

[14] P. Tourón Touceda, S. Mosquera Vázquez, M. Lima, A. Lapini, P. Foggi, A. Dei, R. Righini, *Phys. Chem. Chem. Phys.* **2011**, *14*, 1038–1047.

[15] K. G. Alley, G. Poneti, P. S. D. Robinson, A. Nafady, B. Moubaraki, J. B. Aitken, S. C. Drew, C. Ritchie, B. F. Abrahams, R. K. Hocking, K. S. Murray, A. M. Bond, H. H. Harris, L. Sorace, C. Boskovic, *J. Am. Chem. Soc.* **2013**, *135*, 8304–8323.

[16] G. Poneti, M. Mannini, B. Cortigiani, L. Poggini, L. Sorace, E. Otero, P. Sainctavit, R. Sessoli, A. Dei, *Inorg. Chem.* **2013**, *52*, 11798–11805.

[17] T. E. Fischer, J. T. Janetzki, F. Z. Mohamed Zahir, R. W. Gable, A. A. Starikova, C. Boskovic, *Dalton Trans.* **2024**, 10.1039.D3DT04162J.

[18] L. Yang, M. Dincă, *Angew. Chem. Int. Ed.* **2021**, *60*, 23784–23789.

[19] G. K. Gransbury, B. N. Livesay, J. T. Janetzki, M. A. Hay, R. W. Gable, M. P. Shores, A. Starikova, C. Boskovic, *J. Am. Chem. Soc.* **2020**, *142*, 10692–10704.

[20] G. M. Sheldrick, **1997**, SHELXS-97, Program for Crystal Structure Solution, Göttingen, Germany.

[21] G. M. Sheldrick, *Acta Crystallogr A* **2008**, *64*, 112–122.

[22] G. Bernardinelli, H. D. Flack, *Acta Cryst A* **1985**, *41*, 500–511.

[23] L. J. Farrugia, *J Appl Cryst* **1999**, *32*, 837–838.

[24] M. Llunell, D. Casanova, D. Cirera, P. Alemany, S. Alvarez, **2013**.

[25] N. Suaud, A. Colin, M. Bouammali, T. Mallah, N. Guihéry, *Chemistry – A European Journal* **2024**, *30*, e202302256.

[26] Y. Wang, F. Lambert, E. Rivière, R. Guillot, C. Herrero, A. Tissot, Z. Halime, T. Mallah, *Chem. Commun.* **2019**, *55*, 12336–12339.
